# A precise spatiotemporal fusion crop classification framework based on parcels

**DOI:** 10.1038/s41598-025-03351-7

**Published:** 2025-06-01

**Authors:** Liegang Xia, Changge Chen, Jiancheng Luo, Xiaodong Hu, Xuanzhi Lu, Hongfeng Yu, Keyu Lu

**Affiliations:** 1 College of Computer Science and Technology, Zhejiang University of Technology, Zhejiang, 310023 China; 2 State Key Laboratory of Remote Sensing Science, Aerospace Information Research Institute, Chinese Academy of Sciences, Beijing, 100101 China; 3 School of Information and Electronic Engineering, Zhejiang University of Science and Technology, Hangzhou, 310023 China

**Keywords:** Crop classification, Agricultural phenology, Time series, Synthetic aperture radar (SAR), Object-based texture classification, Computer science, Agroecology

## Abstract

The precise extraction of crop type information on agricultural land supports applications such as agricultural information statistics and planning. It is also a crucial foundation for improving agricultural production efficiency and promoting agricultural informatization. In smallholder agricultural regions, such as the southern agricultural areas of China, a significant number of small parcels exist. These small parcels often exhibit deficiencies and discrepancies in feature representation for time series classification of crop types, leading to considerable classification challenges. To achieve more precise crop type differentiation in smallholder agricultural systems, this study designs a parcel-based classification framework, PITT (Parcel-level Integration of Time series and Texture). The PITT framework categorizes small parcels in smallholder systems by area into small parcels and micro parcels, which are then separately used as inputs for time series classification methods and high-resolution texture classification methods. During the process, the time series classification results guide the high-resolution texture classification method. Finally, the results from the texture classification are fused with the time series classification results, achieving more accurate crop classification outcomes. The study focuses on the Jiang area of Zongyang County, Tongling city, Anhui Province. Experimental validations using Pearson correlation coefficients and TWDTW similarity comparisons reveal that larger parcels have time series features that more strongly represent the features of typical samples. Additionally, when the PITT framework was compared with other time series classification models using real labels, the F1 scores for small parcels of approximately 0.1–0.5 hectares increased for rapeseed and wheat, reaching 0.93 and 0.94, respectively. For micro parcels (less than 0.1 ha), the F1 scores improved by at least 4.11% and 17.05%, respectively. This demonstrates the ability to achieve high crop classification performance with minimal labelling in smallholder systems, advancing the informatization of smallholder agriculture.

## Introduction

Precisely mapping crops with satellite remote sensing images helps the agricultural sector, farmers, and stakeholders better understand, manage, and optimize agricultural activities^1,2^, making it a key technology for achieving precision agriculture^3^. Currently, in the field of crop mapping, medium-resolution remote sensing images are relied upon as the primary data source, and time series classification methods are widely adopted for crop classification. However, in smallholder agricultural areas, especially southern China, owing to the presence of many relatively small parcels, it is difficult to precisely extract temporal features from medium-resolution remote sensing images of these small parcels^4^. Therefore, overcoming this challenge and achieving precise crop mapping has become one of the key challenges that urgently needs to be addressed. To achieve precise crop mapping, the use of medium-resolution and high-resolution remote sensing image data sources in combination with time series methods and other classification technologies is an important optimization solution.

The available methods for classifying crop time series via machine learning include random forests (RFs), support vector machines (SVMs), and decision trees (DTs). Yang^5^ used multispectral Landsat 8 bands acquired from the same season over multiple years and applied RF and SVM algorithms to achieve high-precision crop classification across different years and seasons via deep learning with a recurrent neural network (RNN), a long short-term memory (LSTM-RNN), and a gated recurrent unit (GRU-RNN). More complex models include InceptionTime^6^, TSTransformer^7^, and MiniRocket^8^. These supervised time series models often require more labelled samples to achieve higher classification performance, posing a cost issue concerning sample acquisition. Time series clustering models such as k-Shape (Efficient and Accurate Clustering of Time Series) methods^9^, time series k-means methods based on DTW^10^, and kernel k-means methods^11^ can differentiate time series types without samples, significantly reducing the incurred costs, but their classification performance is often inferior to that of supervised classification approaches. Wang^12^ used two unsupervised learning algorithms, k-means and a Gaussian mixture model (GMM), to cluster Landsat pixels into crop types and concluded that the GMM could precisely classify crop types with appropriate features and low crop diversity. Another classification strategy is semisupervised learning, which connects clustering models and classification models and fine-tunes them with a small number of samples, combining the advantages of both methods, reducing the sample size and performing well in classification tasks^13^. K-Shape is a fast and accurate time series clustering model with scale invariance and shift invariance that captures the similarity between time series curves. InceptionTime is a flexible and efficient deep learning-based time series classification model that employs deep convolutional neural networks to conduct faster and more precise training. The use of the k-Shape and inception time in semisupervised learning reduces costs and yields enhanced classification accuracy.

In smallholder systems, widespread fragmentation and small parcels pose significant challenges for time series classification using medium-resolution remote sensing imagery. Traditional remote sensing classification methods often exhibit limited differentiation capabilities in small parcel scenarios because of the fragmented and irregular shapes of parcels in these systems. On average, parcel sizes range from 0.1 to 0.5 hectares, with a significant number of micro parcels in southern China measuring less than 0.1 hectares. These micro parcels often cover fewer than 3 × 3 pixels in 10 m of Sentinel-1/2 imagery, limiting the extraction of time series features and reducing classification accuracy.

Among existing approaches for improving time series features, time-weighted dynamic time warping (TWDTW) has proven to be highly effective. Belgiu^14^ demonstrated that object-based TWDTW outperforms pixel-based TWDTW. Fu^15^ reported that crop classification accuracy using the TWDTW method heavily depends on the representativeness and proportion, rather than the sheer quantity, of training samples. TWDTW excels in representing crop characteristics in time series curves, making it a valuable tool for improving crop classification performance when representative crop types are carefully selected.

However, most existing feature improvement schemes overlook scale differences in input features. Directly feeding unoptimized features into classification models often results in “negative optimization”^16^. Smaller parcels in smallholder systems contain a greater proportion of mixed pixels, leading to distorted time series feature representation. The time series features of smaller parcels are less representative of typical sample characteristics. Consequently, using parcels of all scales as inputs for time series classification tasks can lead to decreased classification performance because of “feature pollution” in time series data.

To address inaccuracies in medium-resolution feature extraction for small parcels, using higher spatial resolution multispectral remote sensing imagery can effectively improve parcel feature extraction. The addition of high-resolution feature layers has become a common strategy. However, this approach often fails to consider the relationships between features and time series feature differences across parcels of varying scales. This limitation can lead to greater information redundancy in multisource features^17^. To better integrate feature information across resolutions, recent advancements such as HRNet have extended spatial resolution gradients and used majority voting methods to merge multiresolution classification results, achieving higher overall accuracy^18,19^. These methods emphasize the necessity of resolving the inherent differences in feature representation across varying scales to enhance classification performance.

Currently, the crop type classification methods that are applied to high-resolution remote sensing images are mainly object-based deep learning methods. Although pixel-based crop classification is easy to implement and widely used, it cannot link parcel context information when applied to high-resolution remote sensing images and is sensitive to noise^20^. On the other hand, object-based crop classification methods rely on parcel boundaries that have been segmented in advance, using the average, maximum, and median values of all pixels within the parcel boundaries as representative values of parcel features. This approach can reveal high-resolution crop texture features, leading to more precise classification performance^21^. Su and Zhang^22^ combined GEOBIA^23^ with 2 deep learning methods to obtain improved results^24,25^ and applied their method to a crop classification task involving high-resolution remote sensing images, achieving high OA classification performance in small agricultural areas. Object-based deep learning crop classification can be expressed as both semantic segmentation and image classification tasks. The former relies heavily on segmentation quality, whereas the latter has low feature engineering complexity. Therefore, selecting the appropriate image classification method is crucial for a given task. The SPICE (Semantic Pseudo-labeling for Image Clustering)^26^ model is a deep learning image clustering framework based on ResNet18^27^, which can classify images on the basis of semantic similarity and exhibit high-resolution parcel textures when applied to high-resolution parcel images. The SPICE-Self clustering stage can obtain high-confidence texture prototypes for distinguishing parcel textures, providing a new classification feature basis for parcel crop classification.

Inspired by the above background, to achieve more efficient and precise crop type classification, it is necessary to extract and utilize the temporal features of medium-resolution parcels and high-resolution texture features on the basis of parcel-level features. This study designs a classification framework, PITT, that integrates medium-resolution temporal features with high-resolution texture features. The contributions of this study are as follows.

- A scale grading method for irregular agricultural parcels is proposed. This method performs scale classification preprocessing on unmarked arable parcel data, splits out small and micro parcels, and uses the time series data of small parcels as inputs for time series classification and the texture image data of micro parcels as inputs for high-resolution texture classification models.
- A time series classification method, KI (Integration of k-Shape and InceptionTime), that connects the k-Shaped time series clustering model with the InceptionTime time series classification model is designed. The connection involves the use of high-confidence clustering prototypes as inputs for the time series classification model.
- A classification framework, PITT, that integrates the KI time series classification method and the SPICE texture classification model is further developed. The results of KI assist in training the texture classification model, achieving further optimization of the classification performance of smallholder agricultural system parcels.

## Materials

### Study area

**Fig. 1:**
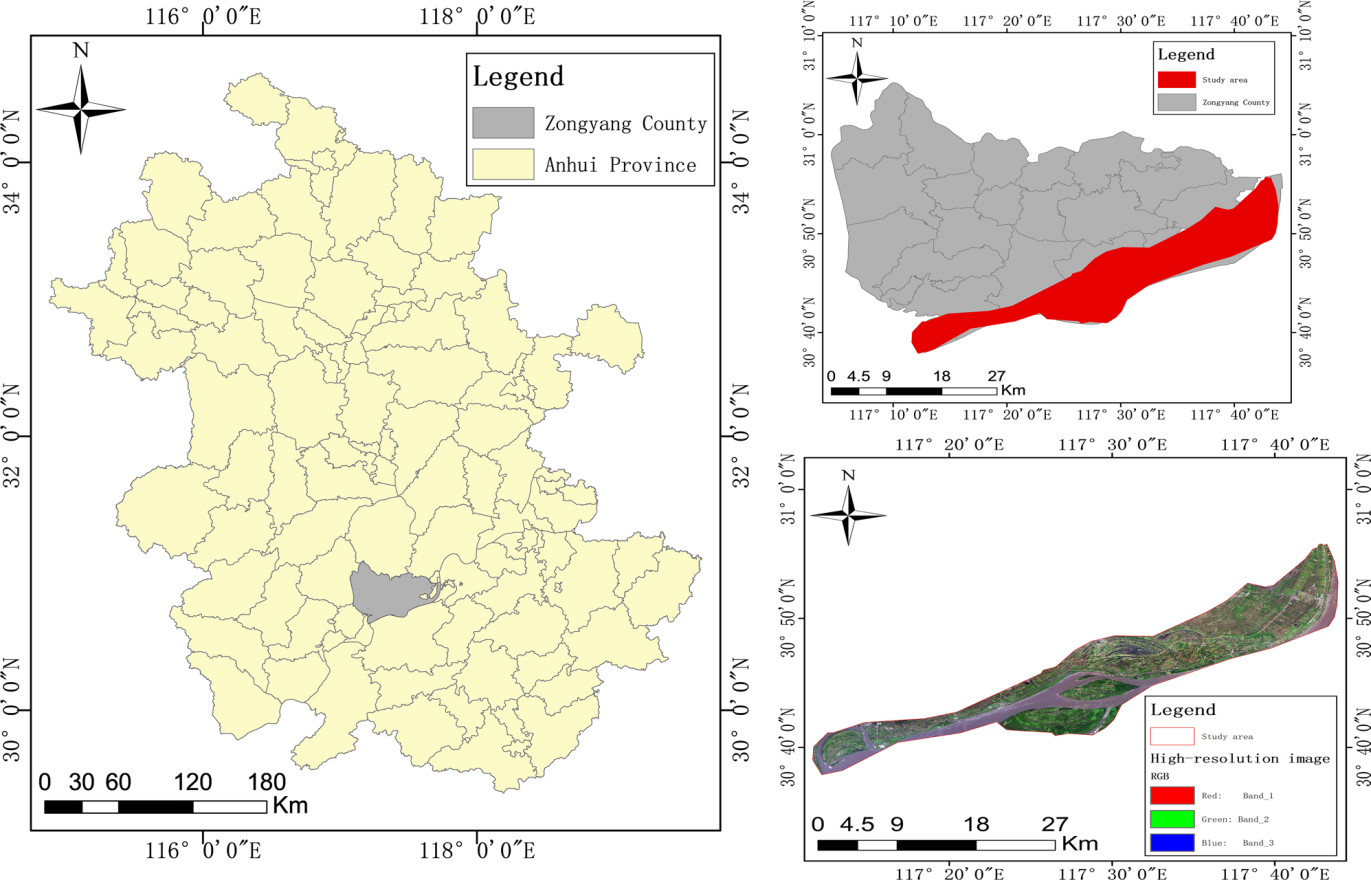
The riverside region of Zongyang County, Tongling city, Anhui Province. {to draw on the software ArcMap 10.8.

The study area is in the riverside region of Zongyang County, Tongling city, Anhui Province, which is the main winter rapeseed and winter wheat production area, as shown in Fig. 1. Zongyang County covers an area of approximately 147,300 hectares, with mountains to the northwest, rivers to the southeast, and numerous lakes within. The main crop types throughout the year are winter rapeseed, winter wheat, double-season rice, and cotton. In the winter and spring seasons from January to May, the main crops produced are winter rapeseed and winter wheat. Other crop types include bare land, wetlands, and grasslands. All parcels have the potential to be replanted with rice, cotton, or corn in April and May during the spring and summer seasons.

Software URL: https://www.arcgis.com/index.html}.

### Satellite data

Tables 1 and 2 show the growth stages of winter wheat and winter rapeseed. Winter rapeseed and winter wheat have similar growing periods, both of which are sown in late October and harvested in late May of the next year.

**Table 1:** Winter wheat phenology in the study area.

Growth periods	Sowing	Seedling	Tillering	Jointing	Tasseling	Mature
Date	Oct.10-Nov.10	Nov.10-Dec.15	Dec.20-Mar.1	Mar.1-Apr.10	Apr.15-May.10	May.15-Jun.1

**Table 2:** Winter rapeseed phenology in the study area.

Growth periods	Sowing	Seedling	Budding	Flowering	Pod	Mature
Date	Oct.1-Nov.10	Nov.10-Jan.20	Jan.20-Mar.10	Mar.15-Apr.10	Apr.15-May.1	May.1-May.15

**Fig. 2:**
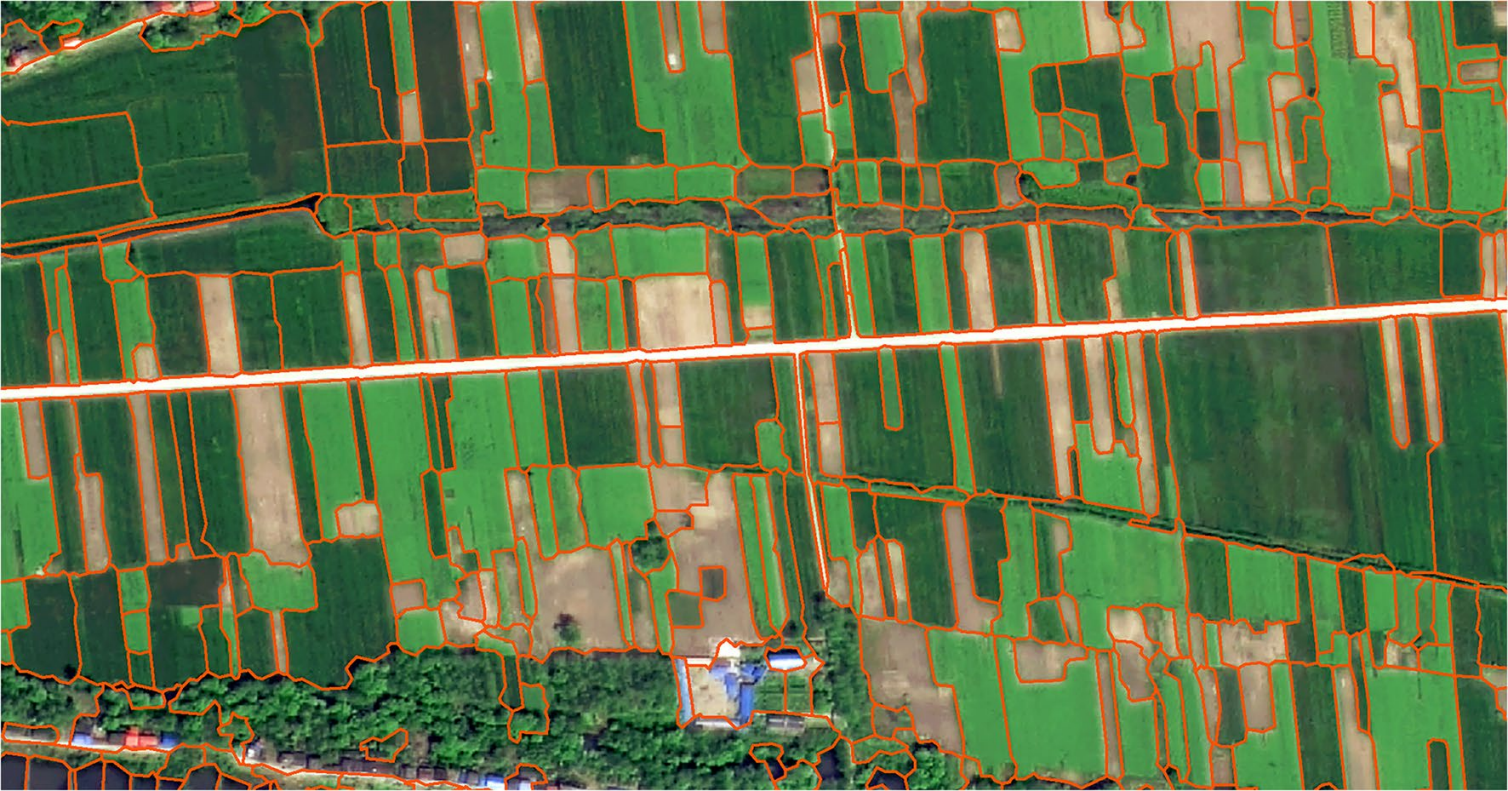
High-resolution image and parcel vector map of the study area. {to draw on the software ArcMap 10.8.

The employed high-resolution remote sensing image is a panoramic remote sensing image of Zongyang County from February 2017, as shown in Fig. 2. At this time, winter rape is in the budding stage, while winter wheat is in the tillering stage, which can distinguish the significant texture features of crops for high-resolution images. This image is stitched together from the GF-2 and BJ satellite images, with a spatial resolution at the submeter level. The image includes three bands, red, blue, and green, which are used as texture classification inputs. The feature extraction method involves capturing square images of a certain scale centred on parcels.

The high-resolution image is accompanied by a panoramic vector map of agricultural parcels. Each parcel in this vector map can be overlaid with high-resolution remote sensing images to extract the image of the parcel and can also be assigned pixel values from multitemporal medium-resolution synthetic aperture radar (SAR) images.

The medium-resolution SAR images acquired by the Sentinel-1 satellite during multiple periods throughout 2017 in Zongyang County. The spatial resolution of the images is 10 m, with only one VH polarization band selected, which mainly covers the Zongyang County area along the river. These SAR images undergo a series of preprocessing steps, such as orthorectification, speckle noise filtering, and radiometric calibration, before they are downloaded from GEE. The time range selected for this batch of SAR images is from January 8 to May 20, 2017, totalling 10 periods that reflect the process of planting to harvest winter rapeseed and winter wheat in Zongyang County. This time includes most of the phenological stages behind winter wheat and winter rape. The time of the most critical phenological stage for crop classification is between mar.1-may.8. Therefore, from the perspective of time series classification, we have fully captured the key phenological stages that are important for classification. The target crops in this study were winter rapeseed and winter wheat. The feature extraction scaling method is based on the parcel-level average, and the SAR-VH band has been verified as the best data source for distinguishing between these two crops^28^.

Software URL: https://www.arcgis.com/index.html}.

### Data analysis

This study considers the following issue: when the same temporal features and time series classifications of crops are used at the parcel level, the different scales and crop types of parcels yield different performances in time series crop classification tasks. The essential reason for this performance difference is that these different scales and types of parcels have different representativeness levels for typical crop types. To analyse these differences, this study proposes a method for measuring the representativeness of parcels with typical crop types: PTR (Phenological Time series Representativeness).

### The impact of parcel scale on PTR

**Fig. 3:**
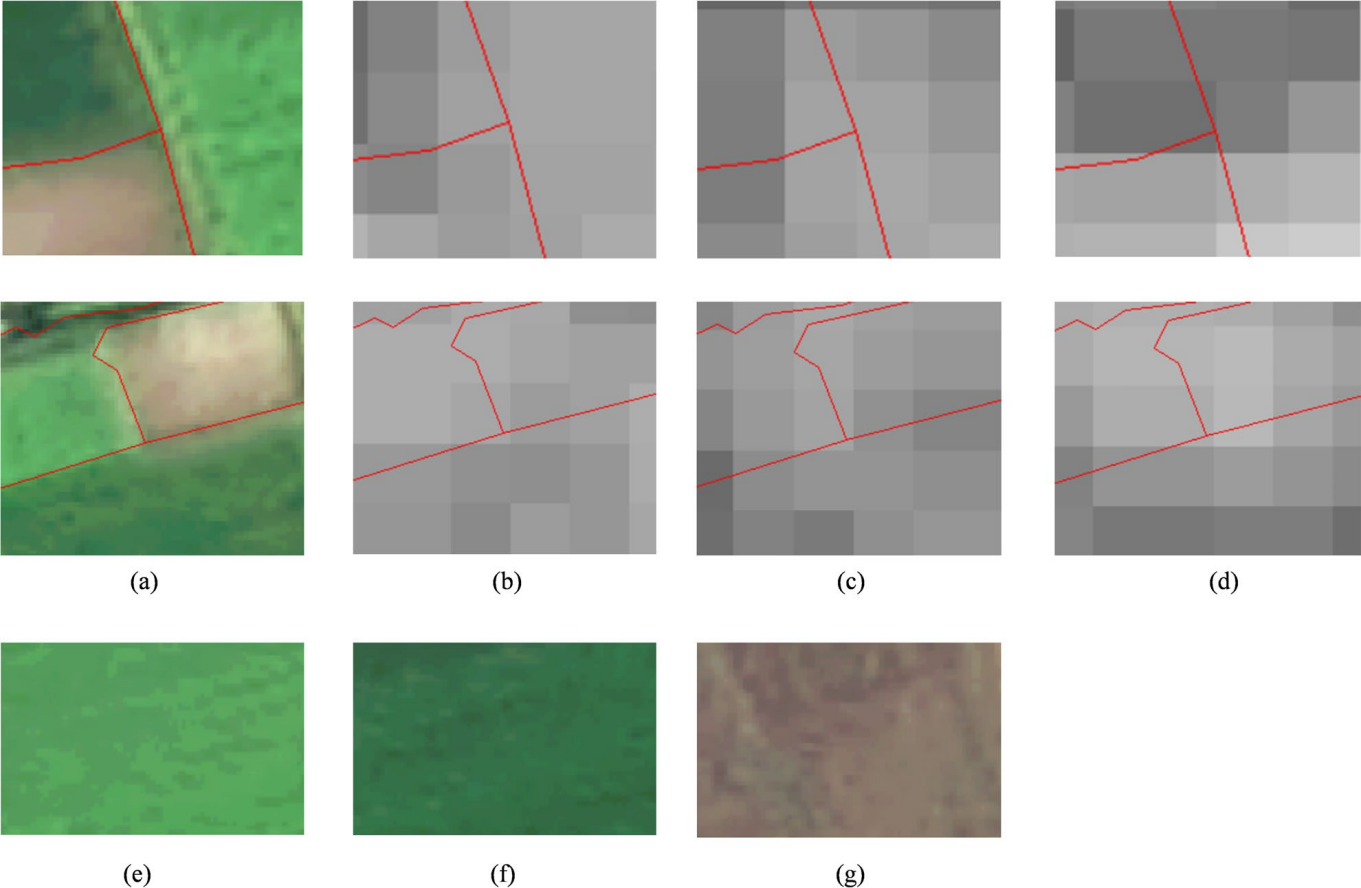
Adjacent parcels with different crop types. (**a**) High-resolution image example of two adjacent parcels with different crop types. (**b**), (**c**), (**e**) SAR-VH images of parcels on different dates. (**e**), (**f**), (**g**) Crop type examples from (a).

The agricultural parcels contained in a region vary in size, and mixed pixels are always present between adjacent parcels, but these mixed pixels are more prevalent in smaller parcels. The adjacent parcels of different crop types in Fig. 3 contain shared mixed pixels, and as time progresses, their temporal feature values change and influence each other. This situation has a greater impact on the temporal feature values of small parcels with at most one medium-resolution pixel than on small parcels, leading to poorer crop classification performance for micro parcels in time series classification methods than for small parcels. This indicates that the temporal feature values of smaller parcels are less representative of typical samples, resulting in a weaker PTR and more negative impacts on the time series classification results obtained for crops.

### The impact of parcel pixel extraction methods on PTR

In the extraction of SAR time-series features for irregular parcels, pixel selection plays a crucial role in determining the accuracy and representativeness of the final features. Object-based feature extraction methods require selecting pixels from within the parcel to reflect its overall characteristics, but the uneven distribution of pixels within a parcel may introduce noise. To address this issue, pixel selection strategies can be categorized into two approaches, as proposed in this study:

#### All-pixel inclusion method

This approach incorporates all pixels within the parcel for computation. It is suitable for cases where the pixel characteristics within the parcel are relatively uniform, effectively capturing overall features.

#### Representative pixel extraction method

This method eliminates boundary pixels or employs denoising algorithms to select internal pixels, reducing boundary effects and noise interference to improve the accuracy of the extracted features.

The all-pixel inclusion method includes edge pixels in time-series feature calculations, which may result in boundary effects affecting the features, particularly in small parcels where the impact is more pronounced. In contrast, for large parcels, the influence of edge pixels is relatively minor due to the high proportion of central pixels, making the all-pixel inclusion method more robust in such cases.

The representative pixel extraction method mitigates the impact of boundary effects by removing edge pixels or selecting central pixels. However, defining representative pixels for large parcels poses a challenge. Simply eliminating edge pixels may fail to account for internal heterogeneity, while selecting only a few central pixels might overlook critical spatial variation within the parcel.

### The impact of SAR polarization types on PTR

**Fig. 4:**
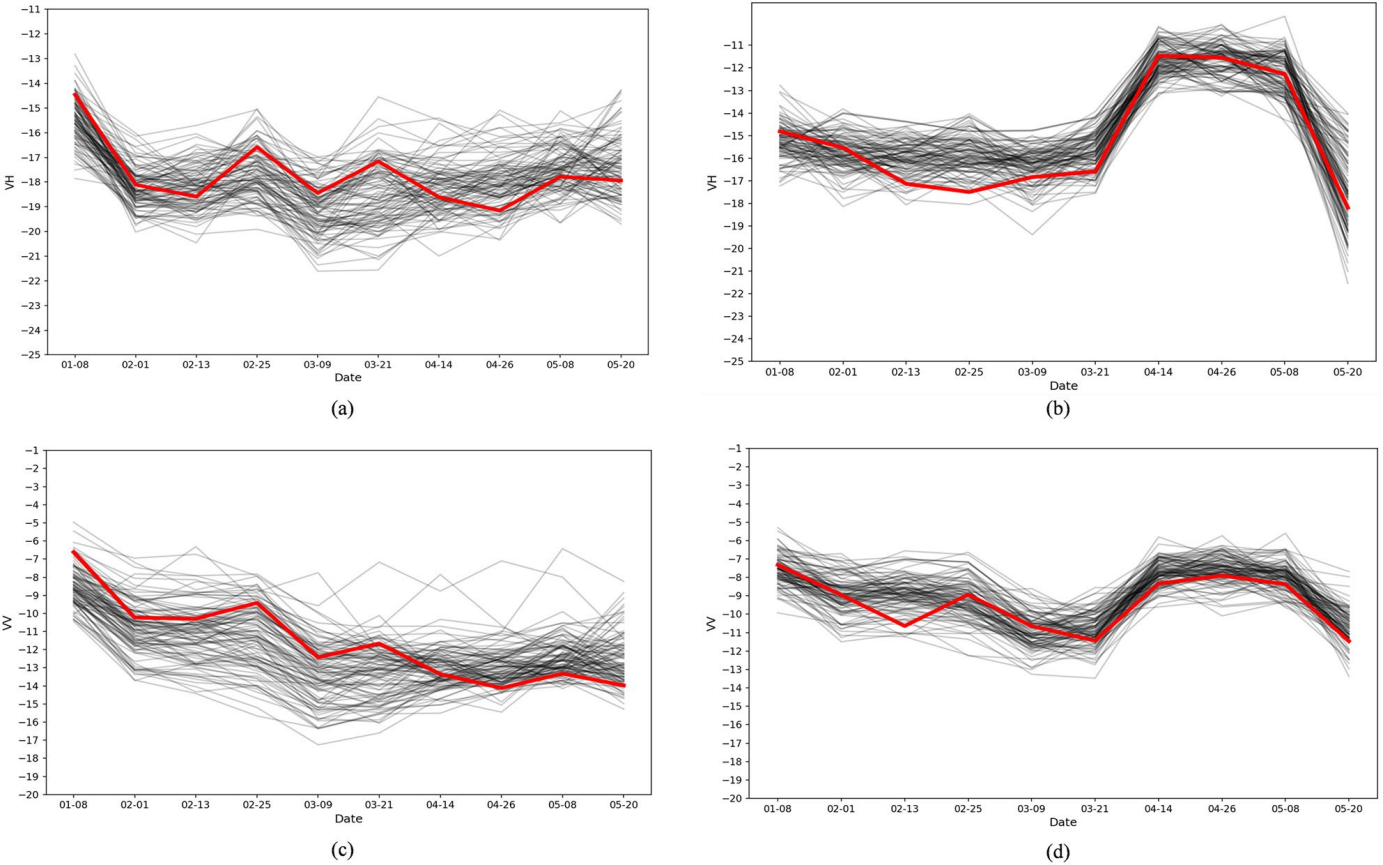
Time series of small parcels. Time series curves for the two crop types, with 100 random real samples selected for each.

Figure 4 illustrate the time-series curves of VV and VH polarization backscatter coefficients for winter wheat and winter rapeseed over time. The dataset consists of real samples from 100 randomly selected large parcels, where black curves represent the backscatter time series of individual samples, and the red curve represents the mean trend.

For VH polarization, the time-series curves of winter wheat and winter rapeseed exhibit distinct seasonal variation trends. The VH polarization values for winter wheat gradually decrease throughout the growth period, showing some fluctuations between March and April before stabilizing. In contrast, winter rapeseed displays a rapid increase in VH polarization, particularly peaking in mid-April before sharply declining. This difference primarily reflects the strong scattering enhancement effect of SAR signals during the bolting and flowering stages of winter rapeseed.

For VV polarization, the winter wheat curve demonstrates a relatively smooth downward trend with lower temporal variation. In comparison, winter rapeseed shows a noticeable increase in VV polarization from March to April, peaking in mid-to-late April before rapidly declining, aligning with the trend observed in VH polarization. This indicates that VV polarization is also highly sensitive to the flowering stage of winter rapeseed, whereas the changes in VV polarization for winter wheat remain relatively stable, mainly influenced by vegetation moisture and structural changes during its growth.

Overall, the SAR time-series curves of winter wheat and winter rapeseed exhibit significant differences under VH and VV polarization. The pronounced scattering enhancement of winter rapeseed in mid-April serves as a key indicator for identifying its critical growth stage. Meanwhile, the stable time series of winter wheat suggests that its key phenological stages can be inferred from the declining trend in VH polarization. These characteristics provide essential references for crop classification and growth monitoring using SAR time-series data.

### PTR representation method

To represent the PTR levels of crops in agricultural parcels, these PTRs are measured by the group average similarity (GAS) and true-average similarity (TAS) metrics, with the Pearson correlation coefficient employed as the similarity measure. For n time series within the group $\{{T}_{1}, {T}_{2}, \dots , {T}_{n}\}$, the similarity *PCC*_*ij*_ among all time series *T*_*i*_ and *T*_*j*_ (including themselves), where 1 ≤ i, j ≤ n, and then the average is taken to obtain the GAS. For n time series within the group $\left\{{T}_{1}, {T}_{2}, \dots , {T}_{n}\right\}$ and a typical sample *T*_*t*_*,* the similarity *PCC*_*i*_ between all time series *T*_*i*_ and the typical sample *T*_*t*_ (including itself) are calculated, where 1 ≤ i ≤ n, and then the average is taken to obtain the TAS.

1 $$GAS=\frac{1}{{n}^{2}}\sum_{i=1}^{n}\sum_{j=1}^{n}{PCC}_{ij}$$

2 $$TAS=\frac{1}{n}\sum_{i=1}^{n}{PCC}_{i}$$

### Data preprocessing by scale

To strengthen the effectiveness of the input of the time series classification method, improve the achieved time series classification performance, and reduce the incurred training time costs, this study proposes a structured representation method for irregular agricultural parcels based on the PTR and preprocesses the parcels according to this method for scale grading.

This method first calculates the radius of the circle inscribed in every parcel and then scales it by deterring mining whether the radius reaches a certain threshold Rt to ensure that the parcel centre contains at least a certain number of nonmixed pixel quantities. Scale levels are established on the basis of the nonmixed pixel distribution of the parcel centre to set scale levels *size*_*i*_, with $i\in \{0, 1, 5, 9, 16, \dots \}$. This method distinguishes smallholder agricultural parcels into small parcels and micro parcels, with micro parcels having areas of less than 0.1 hectares. In 10 m SAR imagery, the number of pixels at the parcel centre is very likely to be less than 5 pixels, so size_5_ is chosen as the standard for distinguishing parcel scales.

**Fig. 5:**
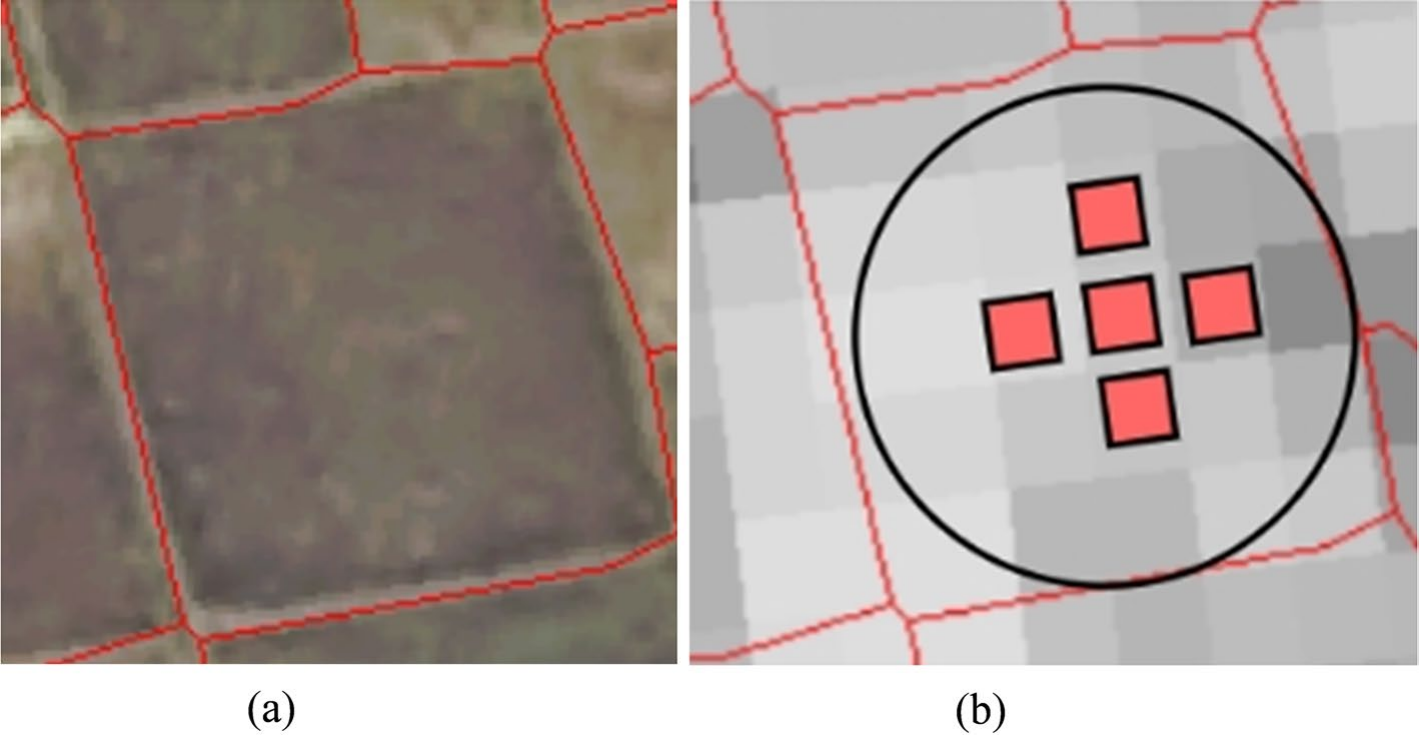
High-resolution images of parcel blocks with at least 5 nonmixed pixels and medium-resolution SAR images. The circles represent the inscribed circles of parcel vector boundaries, with red pixels indicating the central pixels.

**Fig. 6:**
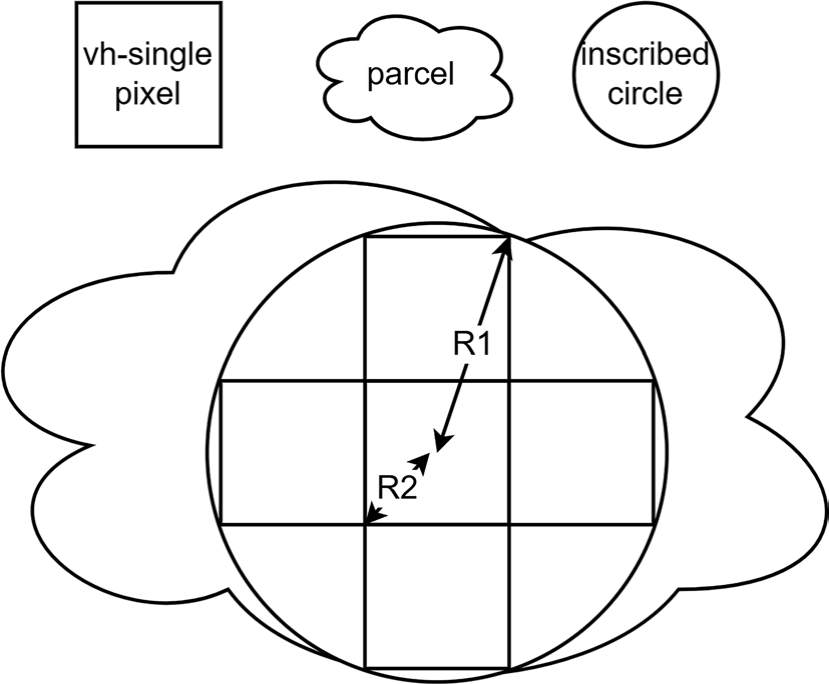
Parcel scale grading-based radius threshold calculation. These central pixels lie within the inscribed circle, demonstrating that when the inscribed circle of a parcel contains more than five pixels, the parcel includes at least five nonedge central pixels.

As shown in Fig. 5, when the radius of the circle inscribed in a certain parcel is ${R}_{ti} \le \text{ R }\le {R}_{tj}$, the scale level of the parcel is *size*_*i*_; when $R={R}_{ti}$, the scale of the parcel is set to the standard *size*_*i*_, with $i<j$, $i\in \{0, 1, 5, 9, 16, \dots \}$ and $j\in \{0, 1, 5, 9, 16, \dots \}$. In Fig. 6, the circle inscribed in the parcel is tangent to 5 nonmixed SAR pixels around the centre, and the centre point of the centre SAR pixel aligns with the centre point of the circle inscribed in the parcel, containing exactly 5 nonmixed pixels. At this point, the parcel scale is the standard *size*_*5*_ scale, and its inscribed circle radius R also represents a radius threshold Rt_5_, with the inscribed circle radius corresponding to a standard-scale inscribed circle radius R_5_. The parcel grading radius threshold *R*_*ti*_, with $i\in \{0, 1, 5, 9, 16, \dots \}$, is calculated via the following method:

3 $${R}_{ti}={R}_{i}+{R}_{b}$$

4 $${R}_{b}={ (\frac{1}{2}L) }^{2}$$

where Ri is the standard radius of the inscribed circle, that is, the parcel centre contains exactly *i* distributed regular nonmixed pixels; $i\in \{0, 1, 5, 9, 16, \dots \}$; Rb is a fixed buffer value that is set to reduce the number 5 of mixed pixels when the centre pixel is not aligned with the centre of the circle inscribed within the parcel; and where L is the fixed side length of each square SAR pixel.

The PTR of crop parcels indicates that among parcels with the same crop type, the temporal feature values of parcels with higher PTR values can better represent all the temporal feature values of the full parcels and can better represent the temporal feature values of typical samples, thereby reducing the impact of lower-quality data on the resulting classification performance. In the comparison among the PTR values of parcels with different scales discussed in Sect. 5.1, the time series data of larger-scale parcels have higher PTR values.

For the above reasons, this study utilizes the medium-resolution time series data of unlabelled small parcels as the inputs of the time series classification method, setting the parcel scale level to *size*_*5*_ and above (refer to Fig. 6), and employs the high-resolution image data of unlabelled micro parcels as the inputs of the SPICE texture classification method, setting the parcel scale level to less than *size*_*5*_.

### Self-made samples

To verify the reliability of the proposed method, an experiment is conducted by preparing several custom-made samples to evaluate the classification effect of the PITT classification framework. In this experiment, approximately 7,000 samples are cross-validated on the basis of the textures of the 2017 high-resolution images and the 2017 VH polarimetric time series curve of the parcels in Zongyang County. These samples are derived from 20 discrete areas along the river, including 16 dryland farming areas, 3 wetland areas, and 1 bare land area. These validation areas reflect the comprehensive classification of the main crops produced in the spring and the nonoutput parcels in the riverside area of Zongyang County. The main crop types during spring include winter rapeseed fields; winter wheat fields; and other parcel crop types, such as dry bare land, grasslands, and wetlands.

**Fig. 7:**
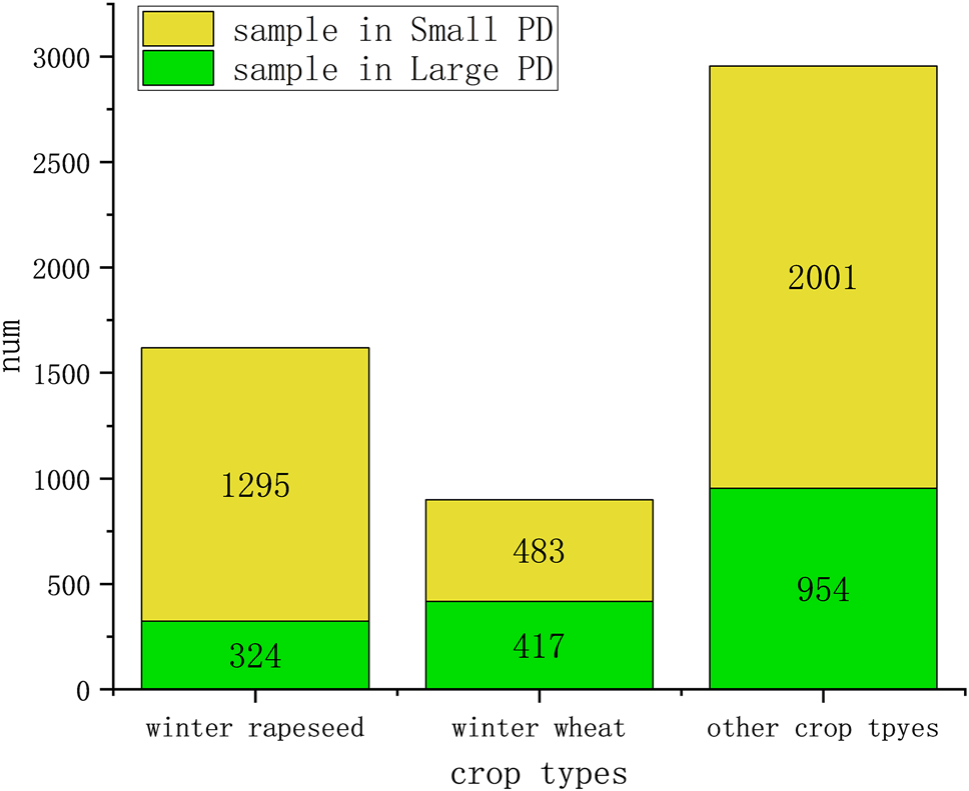
Sample distribution of small and micro parcels with different crop types.

This study labels micro parcels with insufficient size_5_ in the custom-made sample data as the small parcel dataset, whereas larger parcels are labeled as the large parcel dataset, referred to as the small PD and large PD, respectively, with the sample size shown in Fig. 7.

## Methods

**Fig. 8:**
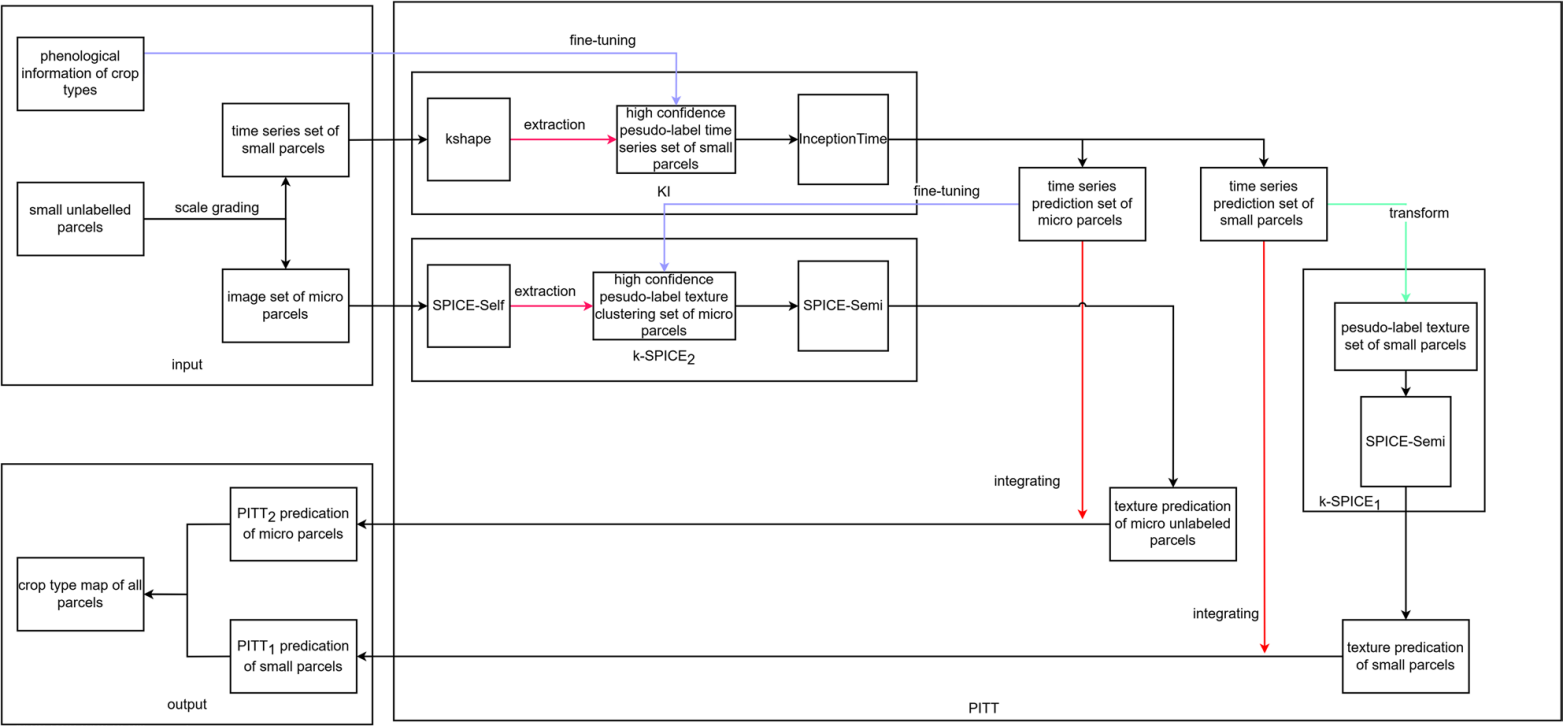
Flowchart of the method.

Figure 8 shows the flowchart of the main methods employed in this work. The main processes are as follows. (1) The KI time series classification method is used to classify the time series data of small parcels, thereby obtaining classification results for both small and micro parcels. (2) Training is conducted with two different high-resolution semantic texture classification inputs for k-SPICE (Intergration of Time Series Model and SPICE), which integrate the KI classification results produced for small and micro parcels, thus obtaining new classification results for both small and micro parcels. (3) Weighted average coefficients are employed to merge the KI classification results and the k-SPICE texture classification results, obtaining the final crop type classification results through PITT.

### Time series classification approach

#### Flow of the time series classification method

For the time series classification task, this study designs a time series classification method called KI that connects the k-Shape time series clustering model and the inception time series classification model. KI first uses the k-Shaped time series model to cluster the time series dataset for small parcels. Then, various time series prototypes are extracted from the clustering results on the basis of the high similarity measure of the k-Shape, and these prototypes serve as pseudolabels for the small parcel time series. Since the clustering results of the time series prototypes represent only the types of time series curves rather than the crop types, the prototypes need to be adjusted using phenological information to determine the crop types for each time series prototype, and exceptionally rare time series prototypes need to be removed. Finally, the adjusted classes are classified and trained via the inception time series classification model, which differentiates among the crop types of the parcels at all the different scales.

### Extraction and fine-tuning of the time series clustering results

KI requires the time series curves of the typical phenological information samples to appropriately extract and fine-tune the k-Shaped time series clustering results so that the clustering results can be effectively classified by the inception time series classification model. Because time series data have different scales and amplitudes, even if all time series curves are limited to a certain range of values, the features at different scales affect the clustering results. Therefore, the time series training sets need to be standardized by the mean to achieve scale consistency across the time series curves when performing time series clustering. In addition, in the clustering results, two curves with similar shapes for the same cluster do not necessarily represent the same crop type because the mean standardization technique employed during preprocessing makes their scales consistent, ignoring the actual VH polarization value differences between the curves, and such differences cause two curves with similar shapes to not belong to the same crop type.

The time series clustering results need to be extracted and fine-tuned through three steps. (i) The curve samples in each cluster are extracted with a similarity threshold based on the distance measure of the k-Shape as high-confidence time series prototype pseudolabels. (ii) The time series curves of different types of time series prototype pseudolabels are compared, and cluster classes with similar shapes but significantly different VH polarization values are filtered out. (iii) The time series curves obtained from the phenological information of typical samples are compared with the time series pseudolabels to determine the crop type for each selected time series prototype.

### The k-SPICE texture classification method integrating the KI time series classification method and the SPICE texture classification model

The k-SPICE texture classification method fuses two parts, namely, the KI time series classification method and the SPICE texture classification model. This approach aims to integrate highly reliable time series features and highly reliable texture features as inputs for texture classification purposes, improving the classification accuracy achieved for spring crop types in small parcels and micro parcels via both texture classification methods.

Considering the differences in spatial resolution between optical and radar imagery, direct image fusion is challenging. Therefore, this study adopts a parcel-based information fusion approach. For radar imagery with a spatial resolution of 10 m, small parcels in smallholder agricultural systems (ranging from 0.1 to 0.5 hectares) typically contain about 3 × 3 to 7 × 7 pixels, whereas micro-parcels smaller than 0.1 hectares contain fewer than 3 × 3 pixels. In parcels with fewer pixels, edge pixels are more prone to forming mixed pixels, incorporating features from outside the parcel and thus distorting the parcel’s time series features when all pixels are used for time series feature extraction.

In contrast, for high-resolution optical imagery (2-m resolution), the number of pixels in micro-parcels is relatively larger than in radar imagery, leading to a lower proportion of mixed pixels and finer pixel-level feature representation.

Based on these principles, this study first categorizes parcels into small parcels and micro-parcels according to their irregular scales. Time series features are extracted from radar imagery for small parcels, while texture features are extracted from high-resolution optical imagery for micro-parcels, followed by texture clustering using SPICE methods.

Through the time series representativeness comparison experiments described in Sect. 4.1, it was found that small parcels, due to their larger area, have more representative and stable time series features compared to micro-parcels. Therefore, the time series features of small parcels are used for training to predict and assign time series feature labels to micro-parcels.

For the first fusion method, k-SPICE_1_, the high-confidence classification results obtained by the KI classifier on small parcels are localized and extracted as inputs for SPICE-Semi texture classification training on high-resolution images. This process yields the high-resolution texture types and corresponding crop types for all small parcels. Finally, the crop type for each small parcel is determined through weighted fusion of texture-based and time series-based classification confidences.

For the second fusion method, k-SPICE_2_, high-confidence texture samples obtained from SPICE-Self clustering on micro-parcels are further screened based on high-confidence time series results. These samples are then used as inputs for SPICE-Semi texture classification training. After training, texture classification types for all micro-parcels are obtained. The final crop types of micro-parcels are determined by weighted fusion of texture-based and time series-based classification results.

### SPICE

The first stage of SPICE, Moco^29^, learns image features through pretraining. In the second stage, SPICE-Self embeds the learned features into the SCAN clustering model to obtain semantic image pseudolabels^30^. Finally, in the third stage, SPICE-Semi uses these semantic image pseudolabels as inputs to fixmatch the semisupervised texture classification model to further improve the texture classification results^31^.

**Fig. 9:**
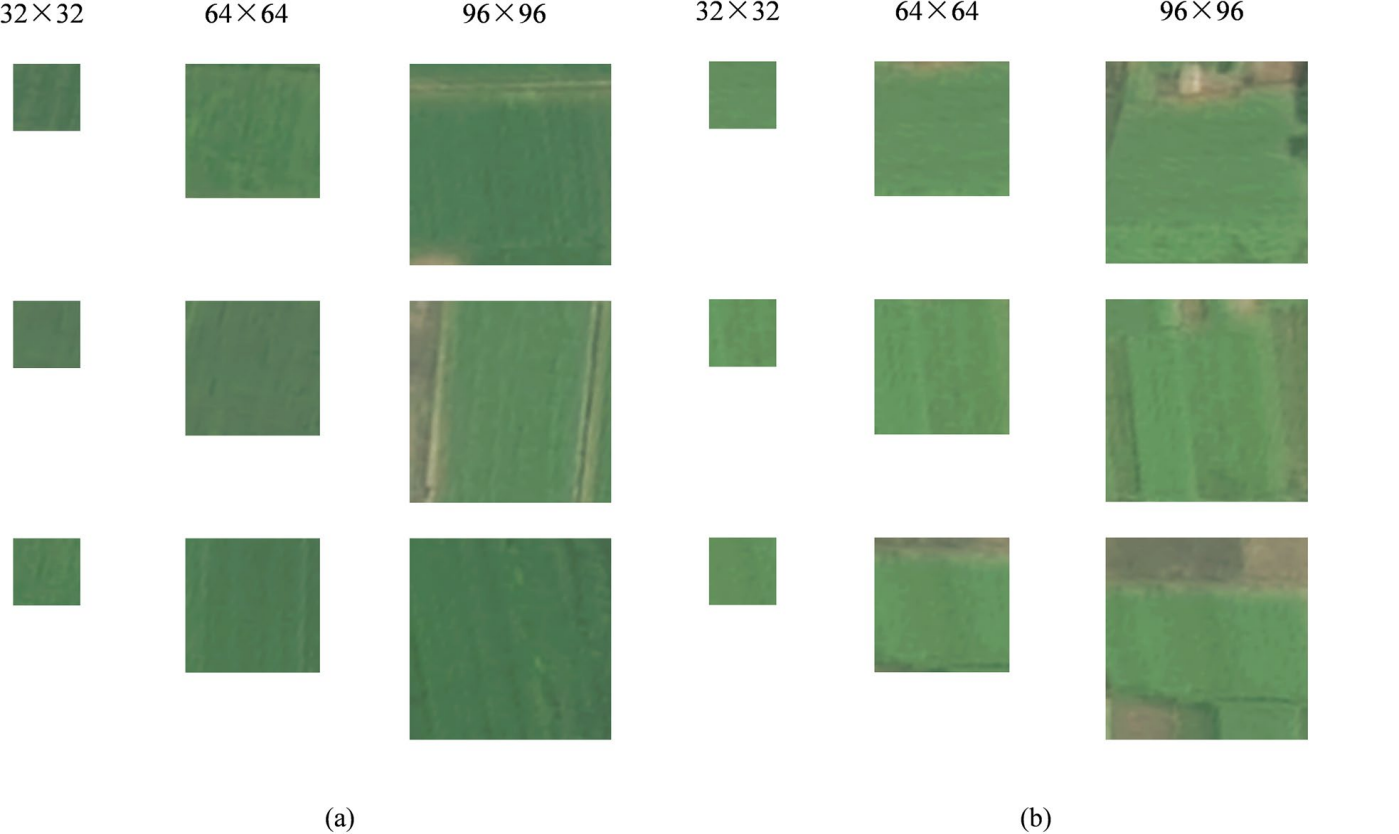
Texture images of winter wheat and winter rapeseed fields at different scales at large PDs.

SPICE uses different adapted backbone networks for input images of different sizes. The backbone network was originally designed for the CIFAR10, Tiny, and STL10 datasets. When data are input during this study, the backbone network can be selected on the basis of the given image size. As shown in Fig. 9, for small parcels, the backbone network used for 32 × 32 images is ResNet18_cifar, whereas for 64 × 64 images, the backbone network is WideResNet_Tiny, and for 96 × 96 images, WideResNet_STL10 is used. For micro parcels, when the image size is smaller than 32 × 32, such as 16 × 16 or 8 × 8, to adapt to the backbone network, these images are resized to 32 × 32.

### *Texture classification k-SPICE*_*1*_

This method selects the parts of the time series in the time series classification task with high credibility, converts them into batch images of different time series types with a certain image size on the basis of the corresponding parcel numbers, uses these images as pseudolabel input data for the time series of the SPICE-semitexture classification model, replaces the input texture pseudolabel dataset for the small parcels, and finally trains to obtain texture classification results for the small parcels.

### *Texture classification k-SPICE*_*2*_

After training and prediction with the SPICE-Self clustering model for micro parcels, the high-confidence texture pseudolabels of the clustering results are extracted. Then, the classification results produced by the KI time series classification method for micro parcels are incorporated to fine-tune the texture pseudolabels as follows. (i) The results are reclassified based on the time series prediction values included within the same texture prototype class. For example, if a texture prototype contains different crops, these crops can be distinguished into more classes. (ii) Images with the same time series prediction label values but low similarity within the same texture prototype are excluded to improve the purity of the connections between the texture prototypes and time series patterns. (iii) Texture prototypes with the same time series prediction labels and similar textures are combined into one texture type.

The SPICE-Semi texture classification model is trained with pseudolabels obtained from fine-tuning as the input dataset, and texture classification results are obtained for micropom parcels.

### The framework PITT, which integrates medium-resolution time series and high-resolution texture classification results

The final step of the PITT framework is a method that fuses the KI classification results and K-SPICE texture classification results via weighted average coefficients. The texture classification methods for small and micro parcels, k-SPICE_1_ and k-SPICE_2_, respectively, use the SPICE classification model, whereas KI uses the InceptionTime model. Therefore, their results cannot be directly fused on the basis of the confidence of the classification results. The KI classification results produced for small parcels and the k-SPICE_1_ results are fused to obtain the PITT classification results for small parcels (referred to as PITT_1_). The KI classification results produced for micro parcels and the k-SPICE_2_ results are fused to obtain the PITT classification results for micro parcels (referred to as PITT_2_). The weighted average fusion method is as follows:

5 $${prob}_{KI}=[{p1}_{rape}, {p1}_{wheat}, {p1}_{other}]$$

6 $${prob}_{k-SPICE}=[{p2}_{rape}, {p2}_{wheat}, {p2}_{other}]$$

7 $${prob}_{PITT}=[{p3}_{rape}, {p3}_{wheat}, {p3}_{other}]$$

8 $${p3}_{rape}=w*{p1}_{rape}+ (1-w) *{p2}_{rape}$$

9 $${p3}_{wheat}=w*{p1}_{wheat}+ (1-w) *{p2}_{wheat}$$

10 $${p3}_{other}=w*{p1}_{other}+ (1-w) *{p2}_{other}$$

In each parcel, each crop type has a probability. The predicted confidence of the KI classification results for a parcel is denoted as *prob*_*KI*_, and the predicted confidence of the k-SPICE texture classification results for this parcel is denoted as *prob*_*k−SPICE*_. We assign weights *w* to the probabilities of the two models, and then the weighted average is calculated to obtain *prob*_*PITT*_. Finally, the crop type of the parcel is determined by the highest value contained in *prob*_*PITT*_.

### Experimental design

The aim of this study is to design a precise crop classification framework, PITT, that integrates medium-resolution time series and high-resolution textures. This framework distinguishes between unlabelled parcel data belonging to small and micro parcels, assigns them to the two input ends of PITT, and integrates and utilizes the medium-resolution time series features and high-resolution texture features of the parcels, aiming to optimize the classification performance achieved for small parcels and improve the accuracy of micro parcel classification. Experiments evaluate the classification performance achieved on the large PD and small PD by the KI, k-SPICE, and PITT classification methods. Owing to the imbalance between the numbers of labelled and unlabelled samples, based on the parcel scale classification strategy, this experiment evaluates the F1 and macro-F1 scores of the k-Shape, KI, k-SPICE, and PITT methods within the overall framework.

11 $$Precision=\frac{TP}{TP+FP}$$

12 $$Recal=\frac{TP}{TP+FN}$$

13 $$F1=2\bullet \frac{Precision\bullet Recal}{Precision+Recal}$$

14 $$mF1=\frac{1}{C}\sum_{c=1}^{C}{F1}_{c}$$

where C represents the number of categories, c represents the cth category, TP denotes the number of true positives, FP represents the number of false positives, FN signifies the number of false negatives, TN represents the number of true negatives, mAccuracy denotes the macroaccuracy, and mF1 represents the macro-F1 score.

## Results and ablation test

**Fig. 10:**
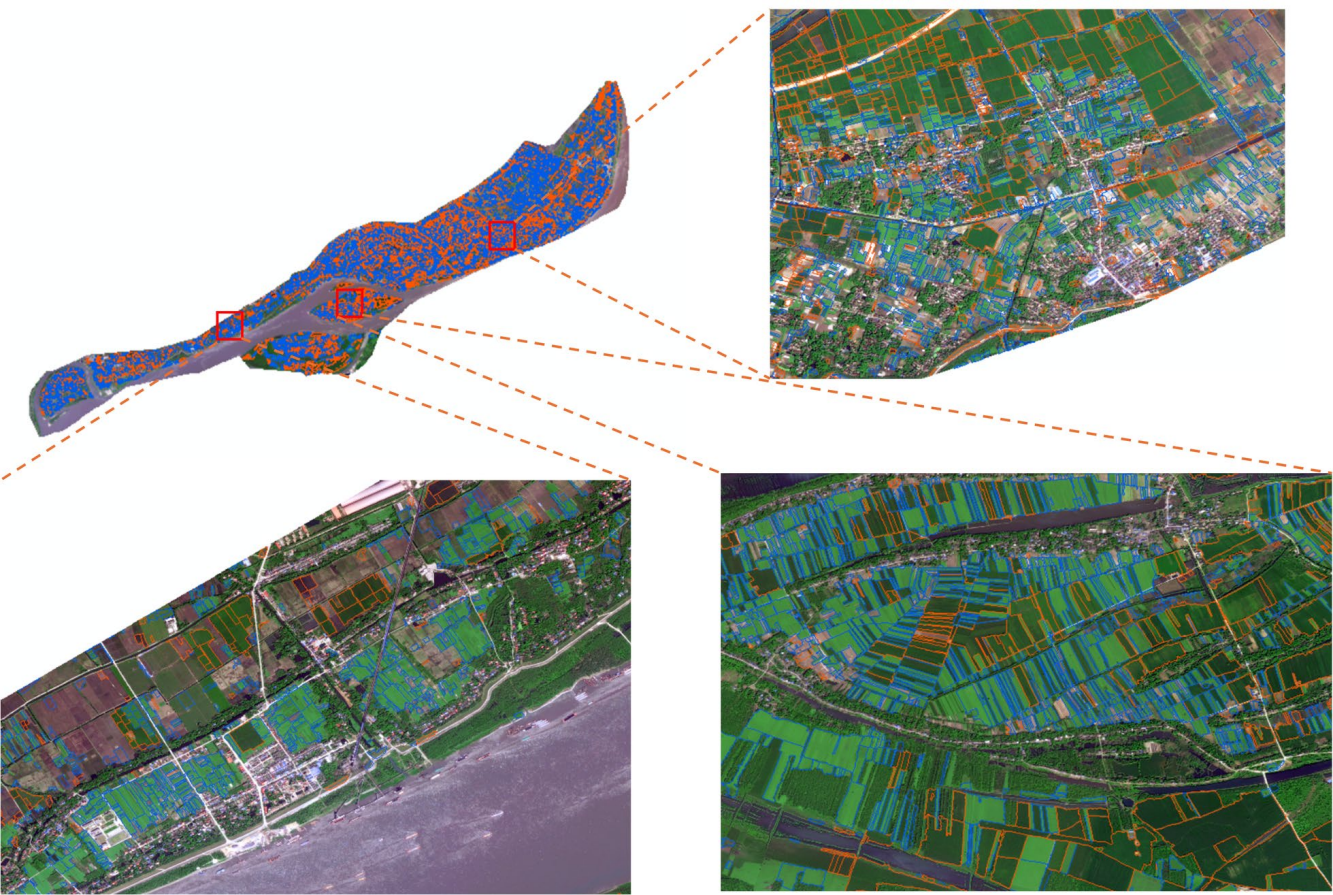
Winter Wheat and Winter Rapeseed Classification Map of the Riverside Region, Zongyang County. {to draw on the software ArcMap 10.8.

Based on the results of this study, crop mapping of winter wheat and winter rapeseed was conducted for the riverside region of Zongyang County, as shown in Fig. 10. In the figure, parcels outlined in orange represent winter wheat, while those outlined in blue represent winter rapeseed.

Software URL: https://www.arcgis.com/index.html}.

### Evaluating the classification performance of the time series classification method

#### Comparison between the classification effects of KI and k-Shape

This experiment validates the effectiveness of using the k-Shaped time series clustering model in the KI time series classification method to connect with the InceptionTime time series classification model to achieve improved time series classification performance. Since clustering lacks generalizability, time series clustering is only used for classifying the Large PD; *prob*_*k−Shape*_ is set to 97% by default. The KI classification results are tested on both the large PD and small PD to demonstrate the differences among the classification performances attained by this method for parcels with different scales.

Table 3 presents the F1-score classification results yielded by the KI time series classification method and the k-square time series clustering model. According to the F1-score classification results produced for the two groups of small parcels, KI improves the classification performance achieved for winter rapeseed by 6.36% over that of k-Shaped rapeseed, whereas the performances attained for winter wheat and other bare land types directly increase by 17.78% and 8.03%, respectively. The mF1 score increases by 10.72%, indicating that the high PTR values of small parcels can significantly improve the classification performance achieved on the Large PD, with the improvement in the F1 score of winter wheat being more sensitive to this combination. A comparison among the classification results produced on the large PD by k-Shape and those produced on the small PD by KI reveals that KI improves the winter rapeseed classification effect by 3.5% and the wheat classification effect by 8.26%, with the mF1 score decreasing by 1.61%. This suggests that the overall low PTR values of micro parcels have side effects on their classification performance, especially for the winter wheat crop type. Overall, larger parcels have greater stability and yield superior time series classification performance because of their higher PTR values, whereas the lower PTR values of micro parcels can have side effects on the time series classification performance attained for these parcels.

**Table 3:** F1-score assessment of the time series classification framework for data with different scales.

Method	Validation data	F1-score			mF1-score
		Winter rapeseed	Winter wheat	Other	
k-Shape	Large PD	0.7667	0.7069	0.8453	0.773
KI	Large PD	0.8303	0.8847	0.9256	0.8802
KI	Small PD	0.8017	0.6243	0.8449	0.7569

### *Comparison between the classification effects of time series clustering pseudolabels with different prob*_*k-Shape*_* values in KI*

**Fig. 11:**
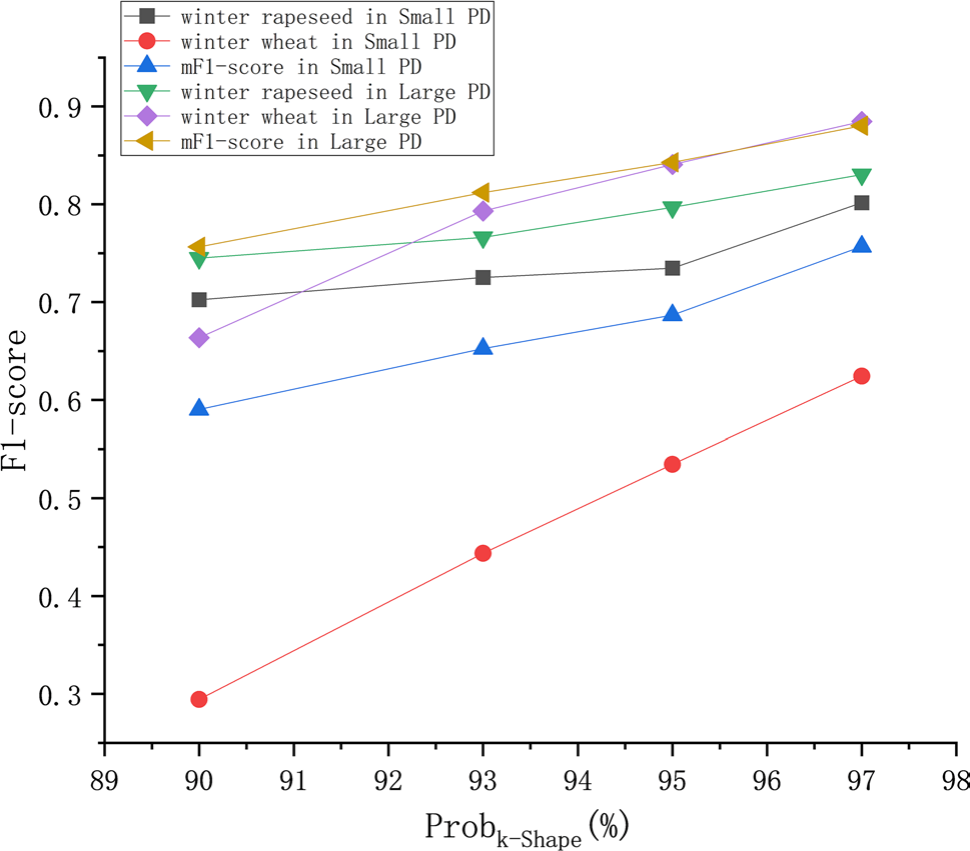
mF1 score assessment of the time series classification results obtained with different _*probk−shape*_ values.

Figure 11 represents the F1-score classification performance of KI when different *prob*_*k−Shape*_ values are used. Since some time series clusters do not have samples when the *prob*_*k−Shape*_ is above 97%, the upper limit for the *prob*_*k−Shape*_ is set to 97%.

A comparison of the classification results obtained for the Large PD and Small PD reveals that the fluctuation range of winter rapeseed with the same *prob*_*k−Shape*_ value is minimal at 2.86%, whereas the fluctuation range of winter wheat ranges from 26.04% to 34.94%. The use of different parcel scales has the least impact on winter rapeseed and the greatest impact on winter wheat under different *prob*_*k−Shape*_ values, which is related to the overall lower PTR of winter wheat. A comparison among the mF1 score classification results produced under different *prob*_*k−Shape*_ values reveals that setting *prob*_*k−Shape*_ to 97% instead of 90% increases the mF1 score by 12.38% for the Large PD and by 16.66% for the Small PD. This indicates that a higher *prob*_*k−Shape*_ value can improve the mF1 score of this time series classification method, especially for micro parcels. In summary, crops with higher PTR values will exhibit more stable classification performance under KI with different *prob*_*k−Shape*_ values, whereas higher *prob*_*k−Shape*_ values will yield better classification performance for KI.

### Evaluation of the classification performance achieved by the PITT framework for small parcels

#### Comparison of the classification effects of KI, k-SPICE and PITT

This experiment validates the feasibility of using the k-SPICE_1_ method for crop type classification on small parcels and confirms the improvement in crop type classification effectiveness provided by the PITT_1_ method for small parcels. These results are compared with the classification results obtained via the KI for small parcels. The image size selected for k-SPICE_1_ in this experiment is 96 × 96, with *prob*_*KI*_ set to 99% in k-SPICE_1_ and *PITT*_*1*_* − w* set to 0.5.

Table 4 shows the F1-score classification results yielded by the three above methods when crop type classification is conducted on the large PD. Compared with the classification results of KI, k-SPICE_1_ decreases the results by 1.11% for winter rapeseed and slightly increases the results by 0.28% for winter wheat. The use of k-SPICE_1_ for small parcels, even when the image clustering stage of SPICE is skipped, can yield crop texture features through the KI results, greatly reducing the training time required for texture learning, with little difference between the achieved classification performance and that of KI. In contrast, PITT_1_ increases the results by 9.6% for winter rapeseed and 5.3% for winter wheat compared with those of KI, with an overall F1-score increase of 6.42%. This indicates that for small parcels, PITT_1_, which fuses parcel time series and texture features via weighted averaging, is superior to classification methods that use time series or texture features alone.

**Table 4:** F1-score assessment of the texture classification methods when conducting time series classification on the Large PD.

Method	F1-score			mF1-score
	Winter rapeseed	Winter wheat	Other	
KI	0.8303	0.8847	0.9256	0.8802
k-SPICE_1_	0.8192	0.8875	0.9266	0.8778
PITT_1_	0.9269	0.9377	0.9686	0.9444

### *Comparison of texture classification performance of k-SPICE*_*1*_* under different image sizes*

In k-SPICE_1_, selecting different image sizes for the image pseudolabels results in different classification performances for small parcels. This experiment compares the texture classification performances achieved for three groups in terms of the F1 scores produced with different image sizes selected for the image pseudolabels.

**Fig. 12:**
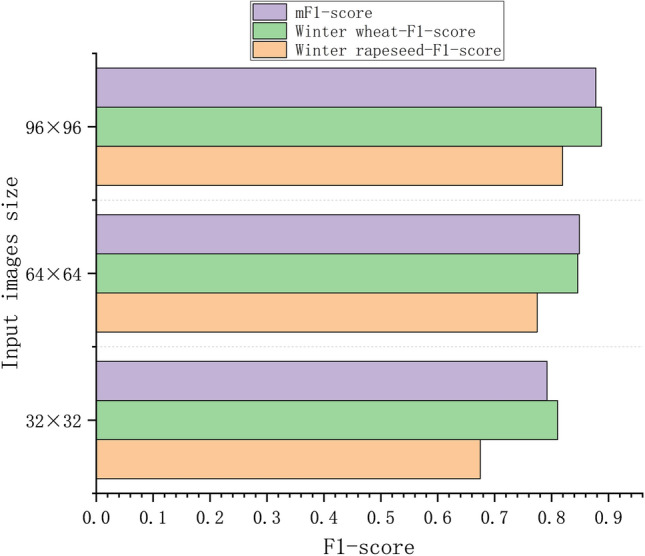
Comparison of the effects produced by k-SPICE_1_ on large PDs under different image sizes.

The F1-score classification results obtained with three different k-SPICE_1_ sizes are shown in Fig. 12. The classification performance of k-SPICE_1_ with an image size of 96 × 96 is the best, with improvements of 14.45%, 7.69%, and 8.56% for winter rapeseed, winter wheat, and the mF1 score, respectively, compared with those obtained when a 32 × 32 image size is used. This finding indicates that for the same KI classification results, choosing a larger and more appropriate image size for the pseudolabels of k-SPICE_1_ enables the crop-type texture features to be more easily captured and significantly improves the resulting texture classification performance. Winter rapeseed exhibited the greatest improvement across different image sizes, with a maximum improvement of 14.45%. The classification effect achieved for winter rapeseed in small parcels is more sensitive to size changes, but the overall F1 score of winter rapeseed is lower than that of winter wheat, indicating that winter wheat can produce better texture extraction effects in small parcels.

### Evaluating the classification performance achieved by PITT for micro parcels

#### Comparison of the classification effects of KI, k-SPICE, and PITT for small parcels

This experiment verifies the feasibility of using k-SPICE for texture classification to improve the classification performance achieved for winter wheat via KI, which has weaker classification results. The experiment compares the classification results obtained by KI, k-SPICE, and PITT for micro parcels. The image size selected for k-SPICE is 8 × 8, that of *prob*_*k−SPICE*_ is 99%, and *w* is set to 0.5.

Table 5 shows the F1-score classification results yielded by KI, k-SPICE, and PITT on the small PD. Compared with the classification results of KI, k-SPICE demonstrates a significant classification performance decrease of 13.02% for winter rapeseed, whereas it provides a 4.29% performance increase for winter wheat. According to the small PD, an overall higher PTR value provides more advantages for winter rapeseed in time series classification tasks than in texture classification tasks. Conversely, an overall lower PTR value makes winter rapeseed weaker in time series classification than in texture classification. Compared with the classification performance of KI, that of PITT slightly decreases (by 1.97%) for winter rapeseed, whereas it greatly increases (by 11.6%) for winter wheat, with a 2.89% increase in the mF1 score. This finding indicates that PITT slightly weakens the classification performance achieved for winter rapeseed and other types but greatly compensates for the negative impact of the overall lower PTR value of winter wheat on the classification performance of the KI.

**Table 5:** F1 score of the assessment of the classification methods on the small PD.

Method	F1-score			mF1-score
	Winter rapeseed	Winter wheat	Other	
KI	0.8017	0.6243	0.8449	0.7569
k-SPICE_2_	0.6773	0.6624	0.7015	0.6804
PITT_2_	0.782	0.7403	0.835	0.7858

### *Comparison of the texture classification performance achieved by k-SPICE*_*2*_* under different image sizes*

In k-SPICE_2_, the use of different image sizes results in different classification effects. To select the appropriate image size as the input for k-SPICE, this experiment compares the F1-score classification performances attained by k-SPICE with different image size inputs. The experiment omits the image clustering stage, using a portion of the samples with three different sizes that are randomly acquired from the Small PD as the inputs of SPICE-Semi, whereas the remaining samples of the Small PD are used as the test dataset, with sizes of 8 × 8, 16 × 16, and 32 × 32; 200 samples randomly selected for each crop type.

**Fig. 13:**
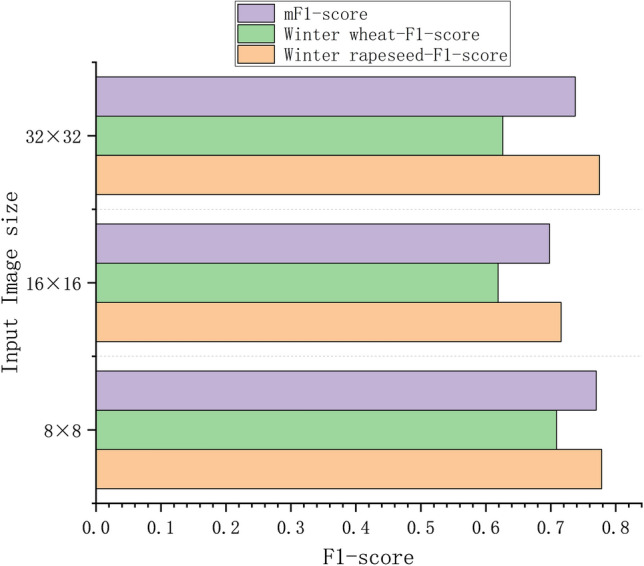
Crop classification results obtained on the small PD after training with image pseudolabels possessing different scales in the SPICE-Semi stage.

The F1-score classification results obtained by k-SPICE_2_ with three different sizes are shown in Fig. 13. Compared with other sizes, when the image size is 8 × 8, the overall F1 scores produced for all crop types are relatively close, and the highest F1 score is achieved for winter wheat. This score is 9.01% higher than that attained with 16 × 16 images and 8.3% higher than that achieved with 32 × 32 images, yielding mF1 score improvements of 7.19% and 3.24%, respectively. Therefore, selecting 8 × 8 images can lead to a more stable improvement in the classification performance of k-SPICE_2_.

## Discussion

### Quantitative comparison of the PTR values of parcels with different feature extraction strategy

To efficiently extract the temporal features of irregular parcels, this experiment conducted a quantitative comparison of PTR under different parcel pixel extraction methods, SAR polarization types, and parcel area scales.

In this experiment on different pixel extraction methods, the study extracted VH-polarized time-series curves and selected 100 random samples of winter wheat and winter rapeseed from parcels classified as size_9_ to compare the PTR performance of different pixel extraction approaches. The representative pixel extraction methods were categorized into the edge excluded extraction and the central pixel extraction. In this experiment, the central pixel extraction method was defined as selecting the central 9 pixels, and the time-series similarity was measured using Pearson similarity.

The results in Table 6 indicate that both the edge excluded extraction method and the full pixel participation method achieved better PTR performance, meaning they more effectively represented the time-series characteristics of crop types. In contrast, the method that only selected central pixels struggled to extract effective crop-type time-series features, suggesting that incorporating more pixels better captures crop-type characteristics. Additionally, the edge-exclusion method demonstrated a slight advantage over the full-pixel participation method for winter rapeseed but a slight disadvantage for winter wheat. This suggests that edge pixels have a greater impact on winter rapeseed than on winter wheat. Therefore, both extraction methods can be considered for experimental reference.

**Table 6:** Comparison of the GAS and TAS values of parcels with different pixel extraction methods. The time series similarity measure used is the Pearson correlation coefficient.

Method	GAS		TAS	
	Winter rapeseed	Winter wheat	Winter rapeseed	Winter wheat
Central Pixel	0.6772	0.3418	0.7869	0.5247
Edge excluded	0.8452	0.5624	0.8667	0.6970
Full Pixel	0.8329	0.5844	0.8591	0.7097

For different parcel pixel extraction methods, this experiment extracted VH polarization time series curves from the Zongyang County dataset and randomly selected 100 samples of winter wheat and winter rapeseed parcels classified as *size*_*9*_ to compare the PTR performance of different pixel extraction methods. The representative pixel extraction methods included the edge-excluded extraction method and the central pixel extraction method. In this experiment, the central pixel extraction method selected the central 9 pixels, and the time series similarity was measured using Pearson similarity.

To further analyze the cross-crop distinguishability between winter wheat and winter rapeseed, when the parameters are set to α = 0.1 and β = 5, the Pearson coefficient for VV polarization is 0.2872, which is significantly higher than that for VH polarization at 0.1778. Additionally, its TWDTW distance is lower at 26.57, indicating that VH polarization can more effectively distinguish the temporal features differences between the two crops. In contrast, VV polarization may be overly sensitive to specific crop features, leading to weaker performance in cross-crop similarity measurement. Therefore, using VH polarization as the source of temporal features is more effective.

Regarding the selection of different SAR polarization types, Tables 7 and 8 present the intra-class average similarity (GAS), typical sample average similarity (TAS), and cross-crop temporal similarity results for different SAR polarization types (VH and VV) in winter rapeseed and winter wheat classification tasks. The experimental results show that for winter rapeseed, VH polarization achieves a GAS of 0.6976 and a TAS of 0.7790, which are significantly higher than those of VV polarization, where GAS is 0.5261 and TAS is 0.5868. This indicates that VH polarization has a stronger capability to characterize the temporal series features of winter rapeseed. However, in the classification of winter wheat, VV polarization exhibits a higher TAS at 0.6128 compared to 0.5261 for VH polarization. Particularly under Pearson coefficient measurement, the temporal similarity with typical samples is more stable for VV polarization.

**Table 7:** Comparison of the GAS and TAS values of parcels with different SAR polarization types. The time series similarity measure used is the Pearson correlation coefficient.

Polarization type	GAS		TAS	
	Winter rapeseed	Winter wheat	Winter rapeseed	Winter wheat
$\text{VH}$	0.6976	0.4033	0.7790	0.5261
$\text{VV}$	0.5261	0.5019	0.5868	0.6128

**Table 8:** The cross-crop distinguishability between winter wheat and winter rapeseed with different SAR polarization types, α = 0.1 and β = 5.

Polarization type	Person coefficient	TWDTW
$\text{VH}$	0.1778	28.25
$\text{VV}$	0.2872	26.57

To further analyze the cross-crop distinguishability between winter wheat and winter rapeseed, when the parameters are set to α = 0.1 and β = 5, the Pearson coefficient for VV polarization is 0.2872, which is significantly higher than that for VH polarization at 0.1778. Additionally, its TWDTW distance is lower at 26.57, indicating that VH polarization can more effectively distinguish the temporal features differences between the two crops. In contrast, VV polarization may be overly sensitive to specific crop features, leading to weaker performance in cross-crop similarity measurement. Therefore, using VH polarization as the source of temporal features is more effective.

This experiment further analyzes the differences in parcel temporal feature representation based on parcel area scale and crop type. A total of 100 random samples were selected from different scales and crop types for grouping. The parcel scale classification standards are set as *size*_*0*_, *size*_*1*_, *size*_*5*_, and *size*_*9*_, and only the winter rapeseed and winter wheat crop types are selected.

**Fig. 14:**
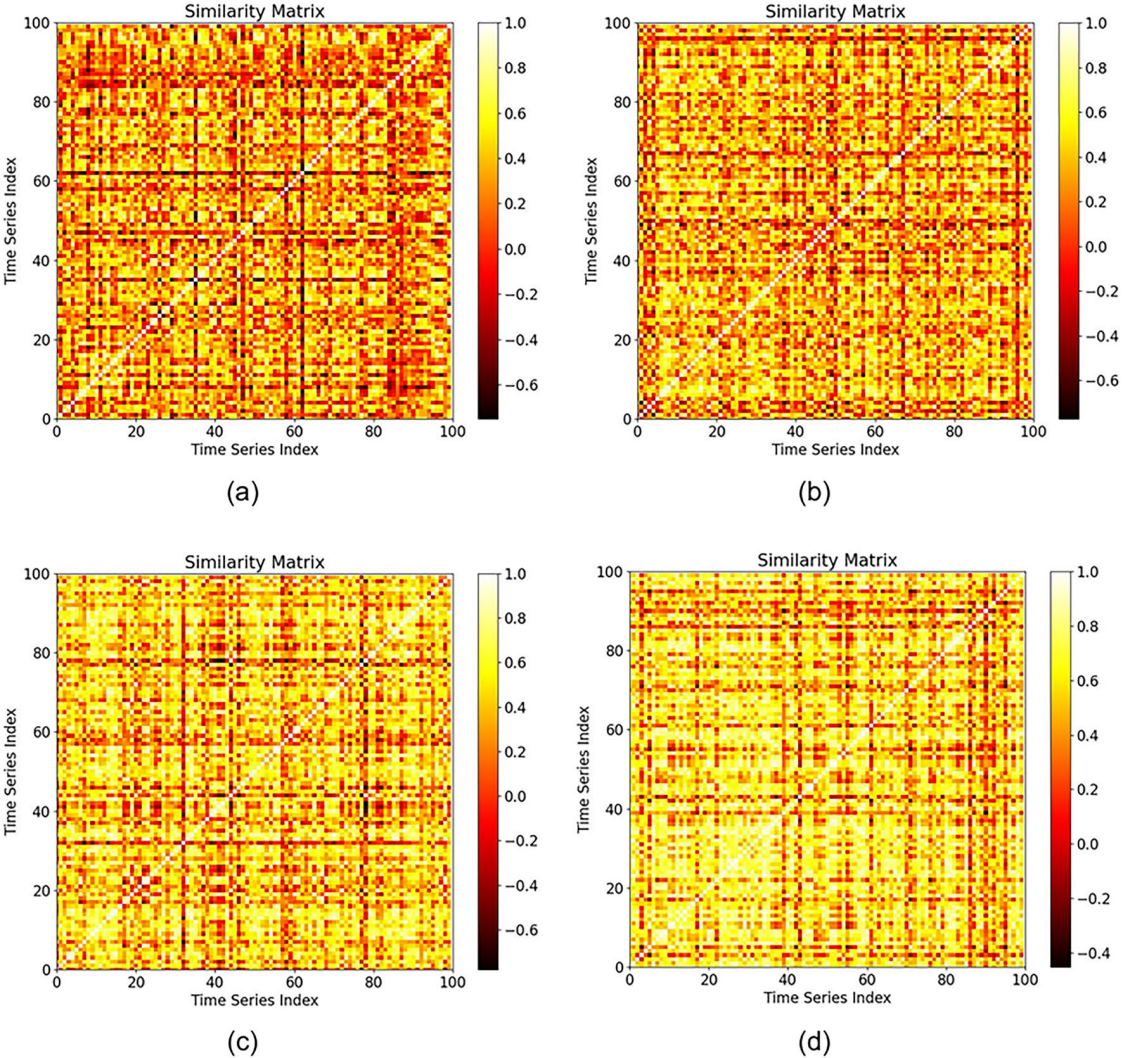
Sparse matrices of the time series similarities among winter wheat groups with different scales.

**Fig. 15:**
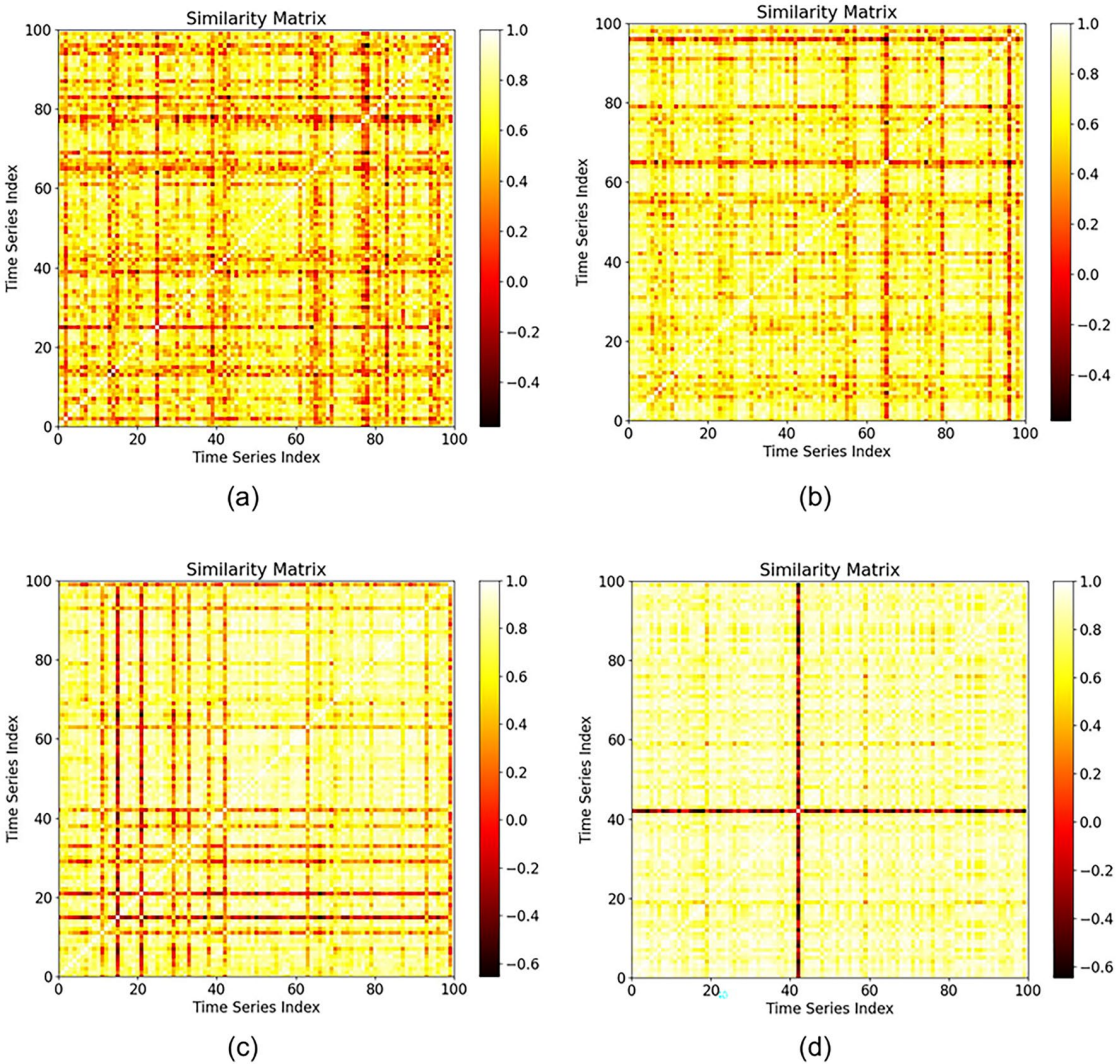
Sparse matrices of the time series similarities among winter rapeseed groups with different scales.

Tables 9 and 10 present the performance results obtained for the PTR according to the GAS and TAS of time series data for parcels with different scales. With increasing parcel scale, the GAS and TAS of each crop type significantly improve, indicating that the time series features of larger parcels with the same crop type have stronger convergence and better represent typical samples. Moreover, when TWDTW is used as the similarity measure, the PTRs of the two crops are similar. However, when the Pearson correlation coefficient is used, the overall GAS and TAS of winter wheat are much lower than those of winter rapeseed, with differences of 24.85% in the GAS and 17.85% in the TAS, respectively, for parcels with a scale of *size*_*9*_. Figure 14 and Fig. 15 represent the sparse similarity with the Pearson correlation coefficient and matrices of the time series curves of winter wheat and winter rapeseed, respectively, which more intuitively show that the overall similarity of winter wheat is weaker than that of winter rapeseed.

**Table 9:** Comparison of the GAS and TAS values of parcels with different scales. The time series similarity measure used is the Pearson correlation coefficient.

Size type	GAS		TAS	
	Winter rapeseed	Winter wheat	Winter rapeseed	Winter wheat
${size}_{0}$	0.5753	0.2773	0.7317	0.3723
${size}_{1}$	0.6737	0.3224	0.8017	0.4476
${size}_{5}$	0.7088	0.4291	0.8236	0.6026
${size}_{9}$	0.8329	0.5844	0.8968	0.7183

**Table 10:** Comparison of the GAS and TAS values of parcels with different scales. The time series similarity measure used is TWDTW, and the parameters used for TWDTW are α = 0.1 and β = 5.

Size type	GAS		TAS	
	Winter rapeseed	Winter wheat	Winter rapeseed	Winter wheat
${size}_{0}$	17.0786	21.4763	20.2123	22.2366
${size}_{1}$	16.4199	18.2477	18.4639	19.5587
${size}_{5}$	15.3772	16.7114	14.6578	15.4905
${size}_{9}$	11.9408	12.8746	12.3592	12.8671

In conclusion, the experimental results validate the critical impact of parcel pixel extraction methods, SAR polarization types, and parcel scales on crop classification performance. VH polarization performed better in cross-crop differentiation tasks and was more effective in finely characterizing winter rapeseed. Additionally, large-scale parcels significantly enhanced the robustness of time series features, providing an important basis for typical sample selection.

### Comparison of the classification effects of different time series clustering models and different time series classification models applied in KI

To demonstrate the performance impacts of different time series clustering models on the KI, this experiment compares the classification performance of the classic k-Shape, DTW-k-means, and kernel-k-means time series clustering models on input time series data derived from the Large PD.

Table 11 shows that DTW-k-Means has a slight advantage over k-Shape and Kernel-k-Means in terms of its classification performance and mF1-score values achieved for all crops. However, KI, which is based on a combination of k-Shape + InceptionTime, results in a greater improvement in crop type classification performance than does DTW-k-Means. Therefore, the keys to improving classification performance lies not in selecting time series clustering models but in choosing an appropriate way to combine these time series clustering models and time series classification models.

**Table 11:** Comparison among the mF1 scores of the classification results produced by different time series clustering models on the Large PD.

Method	F1-score		mF1-score
	Winter rapeseed	Winter wheat	
k-Shape	0.7667	0.7069	0.773
DTW-k-Means	0.7738	0.7161	0.7840
Kernel-k-Means	0.7566	0.6901	0.7436

**Fig. 16:**
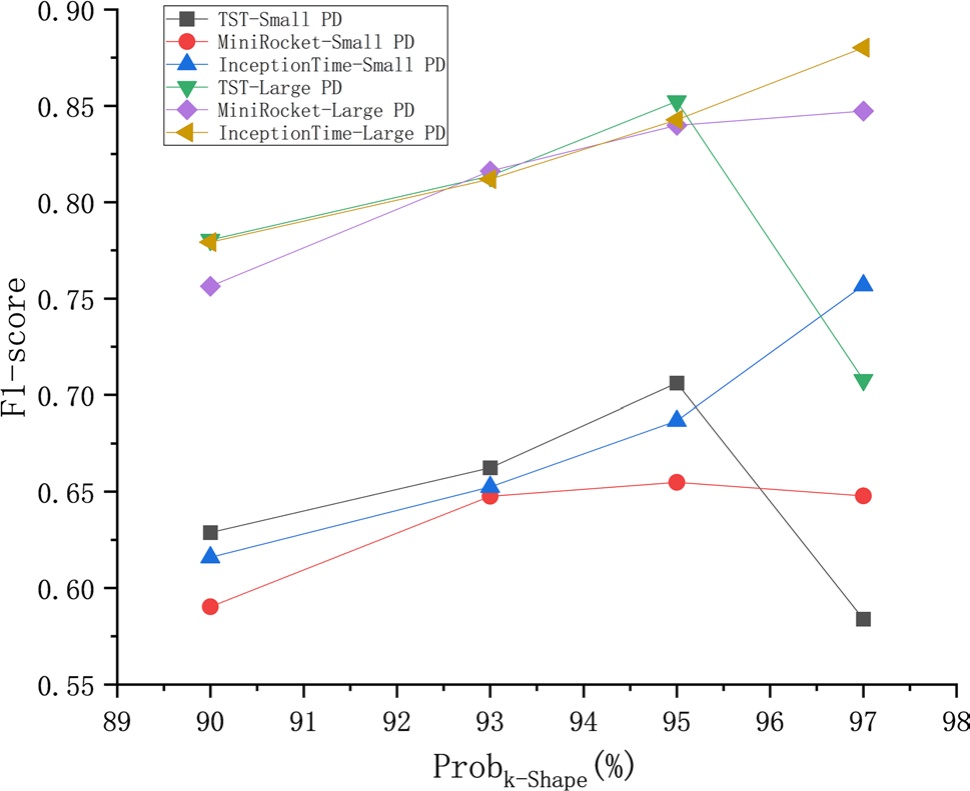
Comparison of the classification results of k-Shape combined with the TST, MiniRocket, and InceptionTime classification models.

In this experiment, the time series transformer (TST), MiniRocket, and InceptionTime are selected as alternative time series classification models, and the *prob*_*k−Shape*_ is set to 90–97% to conduct a comprehensive comparison. As shown in Fig. 16, with increasing *prob*_*k−Shape*_, the InceptionTime group steadily and significantly improves, whereas the MiniRocket group only has a maximum improvement of 3.09% for the Large PD and almost no change for the Small PD. The TSTransformer group performs best when the *prob*_*k−Shape*_ is 95% but sharply declines when the *prob*_*k−Shape*_ reaches 97%.

**Fig. 17:**
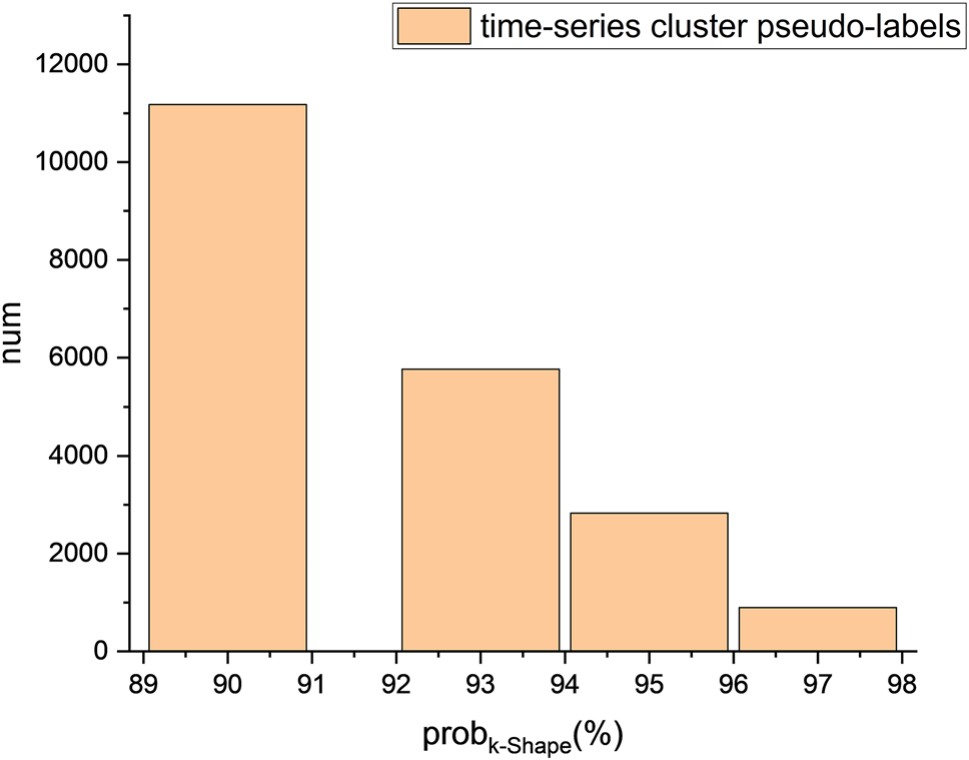
The numbers of time series pseudolabels obtained with different time series clustering confidence levels.

Figure 17 shows the number of time series clustering pseudolabels extracted from different *prob*_*k−Shape*_ values. TST, as a time series classification model, has a high sample input requirement. With *prob*_*k−Shape*_ at 97%, fewer than 1,000 pseudolabels are generated for 97% of the time series, whereas with *prob*_*k−Shape*_ at 95%, 95%of the time series have 2,826 pseudolabels. This reflects the comprehensive constraint effects of the *prob*_*k−Shape*_ and the number of time series pseudolabels on the classification performance of TST, whereas the classification performances of InceptionTime and MiniRocket are proportional to only the *prob*_*k−Shape*_.

MiniRocket, as a time series classification model, has fast and accurate classification capabilities. Its classification effect often converges within fewer training iterations, but this also results in small variations in the classification results, and the overall mF1 score achieved on the small PD is lower. On the other hand, the InceptionTime model is flexible and efficient, with a 97% *prob*_*k−Shape*_ value achieving the highest mF1-scores for InceptionTime. These scores are 88.02% for the large PD classification results, which is 3.29% higher than the highest values of MiniRocket and TST, and 75.69% for the small PD classification results, which is 10.2% higher than the highest values of MiniRocket and TST. Compared with other methods, choosing InceptionTime as the time series classification model for KI yields the best crop classification performance for small parcels with higher *prob*_*k−Shape*_ values, and InceptionTime requires the smallest number of pseudolabel inputs for time series clustering.

### Selection of the weighted average coefficient in the PITT

PITT combines the classification results of k-SPICE and KI through weighted average coefficients to obtain fused classification results for time series textures, and the classification effect is influenced by the selection of the weighted average coefficients *w*. To select the most suitable *PITT*_*1*_* − w* and *PITT*_*2*_* − w* values, this experiment compares their F1-score classification results when varying from 0.051. Among them, the *prob*_*k−Shape*_ is selected as 97%, the *prob*_*KI*_ is selected as 99%, the image size of k-SPICE_1_ is 96 × 96, and the image size of k-SPICE_2_ is 8 × 8.

**Fig. 18:**
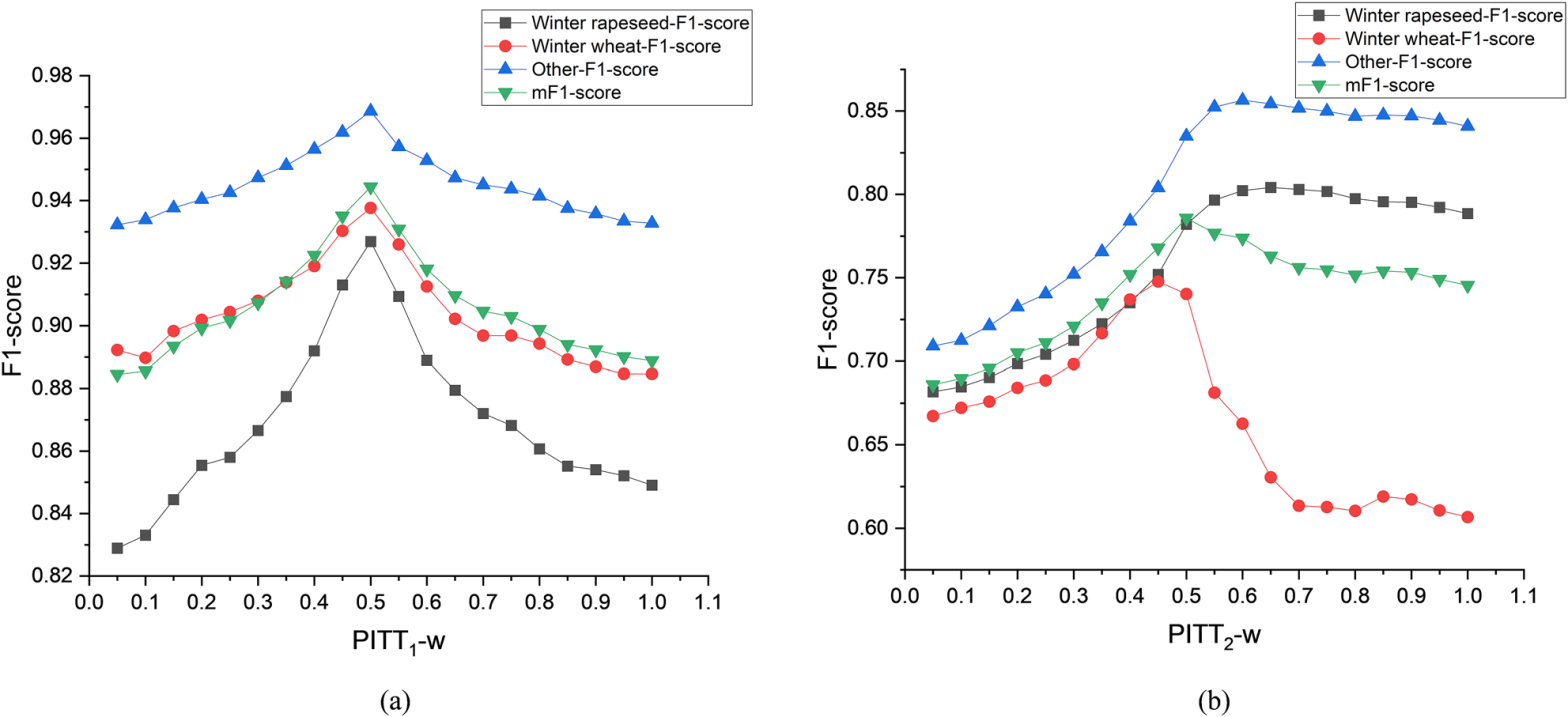
F1-score classification results produced by PITT under different weighted average means *w*.

As shown in Fig. 18a, for the classification of all crop types in the large PD, the optimal choice for *PITT*_*1*_* − w* is 0.5. One possible reason for this is that the results of k-SPICE_1_ are obtained by transforming the KI and texture classification results, and both have very similar F1 score-based classification effects on small parcels. This makes k-SPICE_1_ and KI have the same constraint effect on the classification performance of PITT_1_. The classification effect of winter wheat fluctuates the most due to the influence of *PITT*_*1*_* − w*, reaching a difference of 9.8%. This means that the overall lower PTR of winter wheat leads to instability in the classification performance of the KI and indirectly causes instability in the classification performance of k-SPICE_1_ with respect to winter wheat.

Figure 18b shows that the best classification effect achieved across all crop types in the small PD may not necessarily have a *PITT*_*2*_* − w* of 0.5. However, in terms of balance, selecting a *PITT*_*2*_* − w* of 0.5 can yield the highest mF1 score of 78.58%. When *PITT*_*2*_* − w* is 0.65, the classification result obtained for winter rapeseed reaches the highest value of 80.42%, whereas when *PITT*_*2*_* − w* is 0.6, the highest classification result of 85.65% is achieved for the other types. On the other hand, choosing *PITT*_*2*_* − w* as 0.45 can provide the highest classification result of 74.77% for winter wheat, indicating that the time series features play a larger role in the classification results of winter rapeseed and other types, whereas the image texture features play a larger role in the classification results of winter wheat. This is also reflected in the comparison between k-SPICE_2_ and KI in terms of their classification effects on the small PD.

### Comparison between the classification effects of PITT and InceptionTime

To highlight the superior performance classification of PITT, this experiment compares the classification results of the time series classification models InceptionTime, MiniRocket, and TST with those of PITT on both large PD and small PD. In this experiment, the input of the time series classification models consists of 200 samples of various crop types randomly acquired from the Large PD, whereas the remaining samples in the Large PD and Small PD are used as the test dataset for time series classification models. The input of PITT consists of unlabelled data with a scale of *size*_*5*_ or above, with the Large PD serving as the test set for PITT_1_, the *prob*_*k−Shape*_ set to 97%, the *prob*_*KI*_ set to 99%, and the image size of k-SPICE_1_ being 96 × 96.

**Fig. 19:**
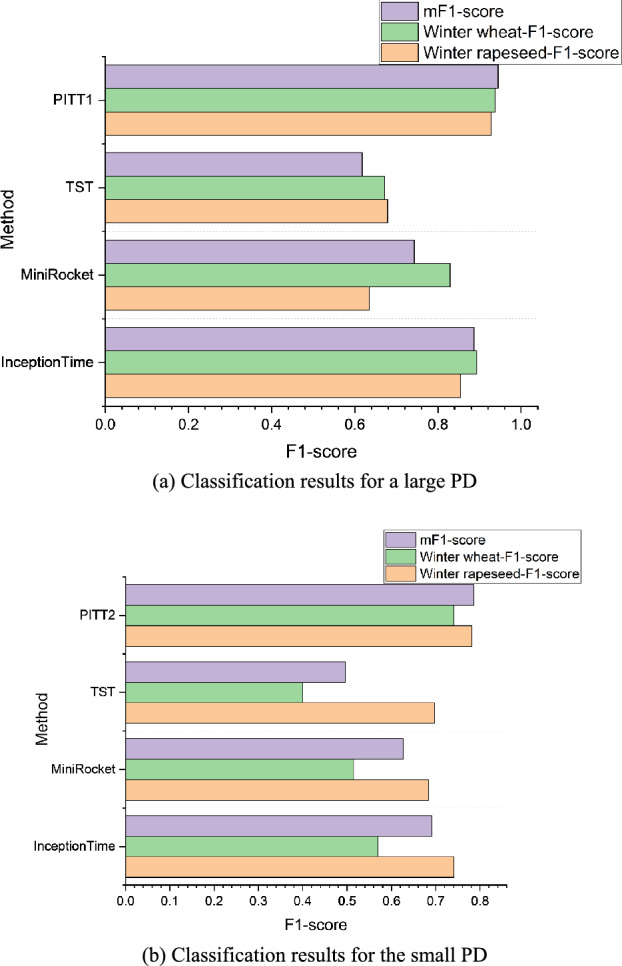
Comparison between the F1-scores of the classification results produced by other time series classification models and PITT.

Figure 19 presents a comparison between the F1-score classification results of the PITT and time series classification models. The Large PD achieves improvements of at least 7.33%, 4.44%, and 5.8% for winter rapeseed, winter wheat, and the mF1 score, respectively. Similarly, the small PD achieves improvements of at least 4.11%, 17.05% and 9.43% for the same metrics. This indicates that PITT can achieve better classification performance with only a small number of typical samples. Compared with the use of time series features alone, the use of both the texture features of crop parcels and time series features for classification and fusion can lead to superior classification performance.

### Limitations

Owing to the high cost of acquiring high-resolution multispectral remote sensing images for commercial purposes and the fact that only high-resolution image data for Zongyang County from February 2017 are used herein, this study can cross-validate only the winter wheat, winter rapeseed, and other crop parcels in the study area during the winter and spring of 2017 for an authentic examination. It is not possible to cross validate summer corn and double-season rice or other winter and spring crops from different years. However, if multiple high-resolution SAR images can be obtained for the current season, the proposed method can still classify them in time series. High-resolution multispectral remote sensing images often cover smaller areas than medium-resolution remote sensing images do; thus, PITT can achieve crop type mapping in only a small area, and in this experiment, crop type mapping is carried out in the riverside area of Zongyang County.

In fact, PITT requires very few typical samples and some unlabelled crop parcels, greatly reducing the sample cost; however, PITT typically trains the k-Shaped time series clustering model and the SPICE texture classification model, which generates more training time costs than standalone time series classification models such as InceptionTime and TST do.

## Conclusion

This study designs a classification framework, PITT, based on the fusion of medium-resolution temporal features and high-resolution texture features. The framework splits the input data into different parcel scales for training the KI time series classification model and the k-SPICE high-resolution classification model in a step-by-step manner and finally merges them to obtain the results of PITT. The experiment validated the classification performance by splitting custom-made samples from the smallholder agricultural parcel research area into large PD and small PD for small and micro parcels. The results show that the temporal features of larger parcels can better represent the temporal features of typical samples and using them as the inputs of time series classification methods can reduce the “contamination” of data from micro parcels during time series training. The PITT method fuses the medium-resolution temporal information and high-resolution texture information of parcels. For small parcels, compared with the InceptionTime time series classification model, PITT improves the mF1 score by 5.8%. For micro parcels, PITT improves the F1 score by 11.6% over that of KI for winter wheat, demonstrating that the PITT framework, which integrates medium-resolution temporal information and high-resolution texture information, further optimizes the classification performance achieved on small parcels and compensates for the poor time series classification performance attained for winter wheat in micro parcels owing to its low overall PTR, thereby benefiting precision agriculture. However, PITT has certain limitations. PITT relies on very few typical samples as cue information and requires more training time for time series clustering and image clustering tasks, which increases the incurred training costs. PITT uses a weighted average-based fusion method, which can be further improved in the future to enhance the effect of fusing temporal and textural features.

## Supplementary Information

Supplementary Information 1.

Supplementary Information 2.

Supplementary Information 3.

Supplementary Information 4.

Supplementary Information 5.

Supplementary Information 6.

Supplementary Information 7.

Supplementary Information 8.

Supplementary Information 9.

Supplementary Information 10.

Supplementary Information 11.

Supplementary Information 12.

Supplementary Information 13.

Supplementary Information 14.

Supplementary Information 15.

Supplementary Information 16.

Supplementary Information 17.

Supplementary Information 18.

## Data Availability

All data generated or analysed during this study are included in this published article [and its supplementary information files.
