# Supplementary material for: A precise spatiotemporal fusion crop classification framework based on parcels
Source: Sci Rep. 2025 Jun 1;15:19208. doi: 10.1038/s41598-025-03351-7 (PMC12127452; doi:10.1038/s41598-025-03351-7)

1. High resolution map of Zongyang County


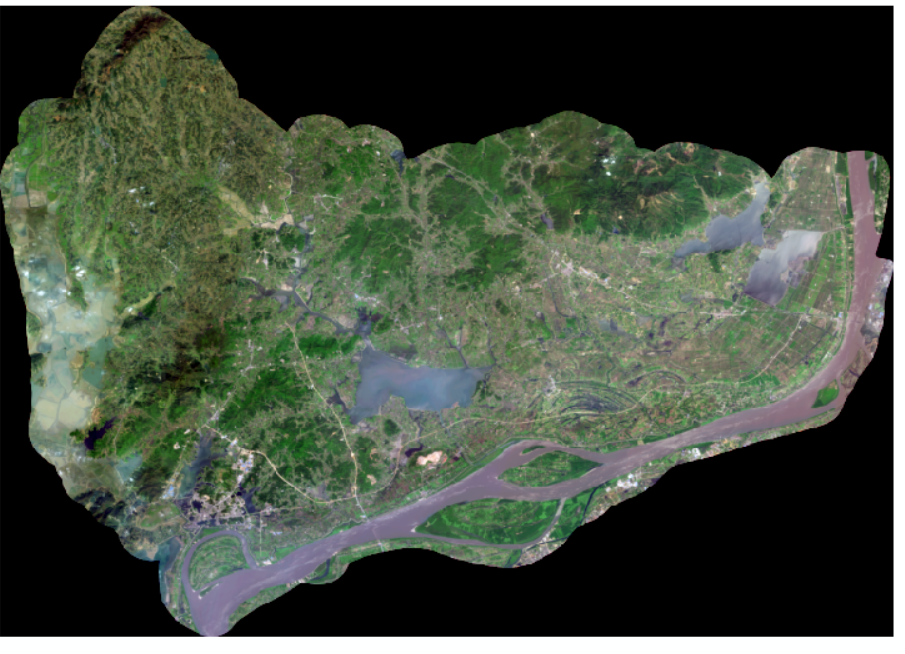


2. High resolution partial map of Zongyang County


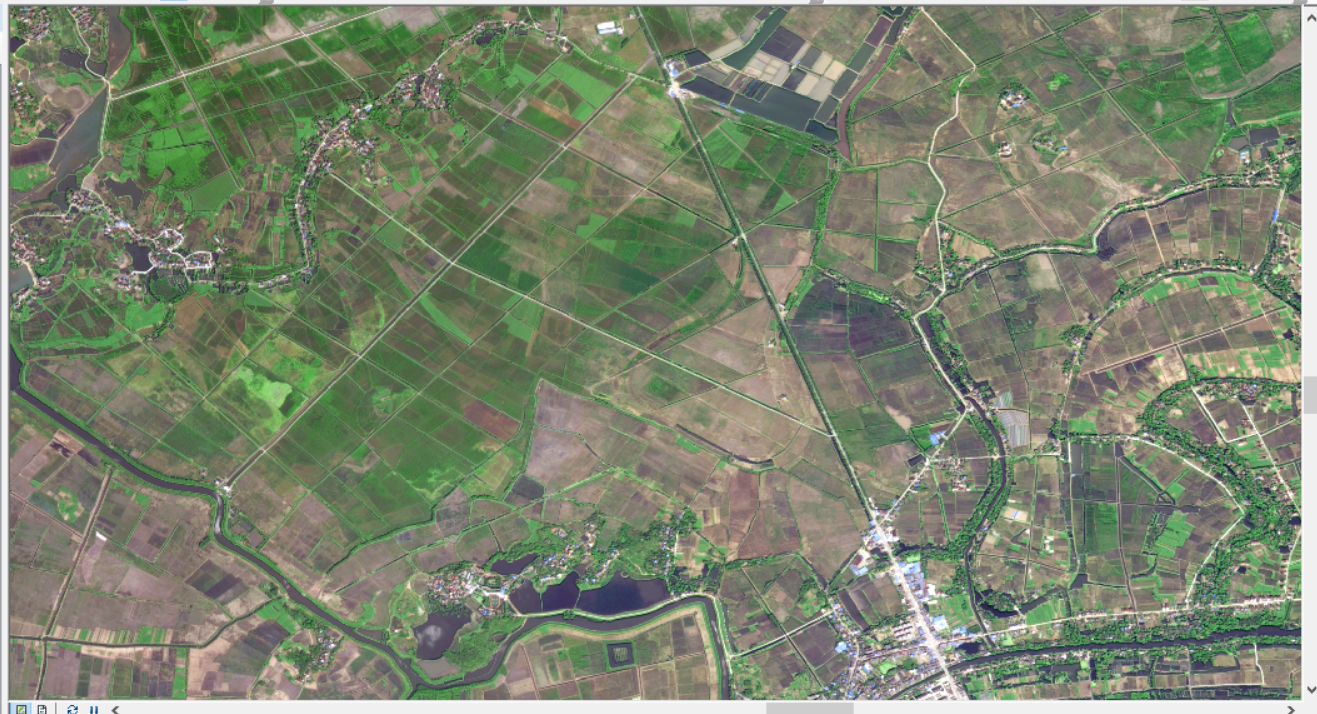


3.Study area vector map and high resolution map of the area along the river in Zongyang County


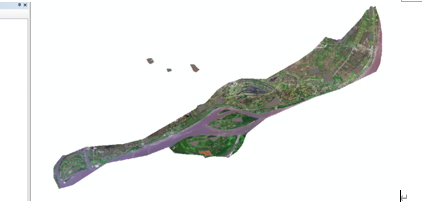

Supplement: Supplementary file 12 — Supplementary Information 12. [file 41598_2025_3351_MOESM12_ESM.docx]
