# Supplementary material for: A precise spatiotemporal fusion crop classification framework based on parcels
Source: Sci Rep. 2025 Jun 1;15:19208. doi: 10.1038/s41598-025-03351-7 (PMC12127452; doi:10.1038/s41598-025-03351-7)
Supplement: Supplementary file 15 — Supplementary Information 15. [file 41598_2025_3351_MOESM15_ESM.docx]

1.Various types of graphs of high reliable results of K-shape clustering


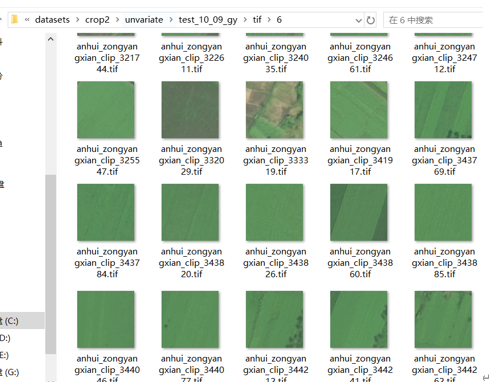


1. Winter rapeseed


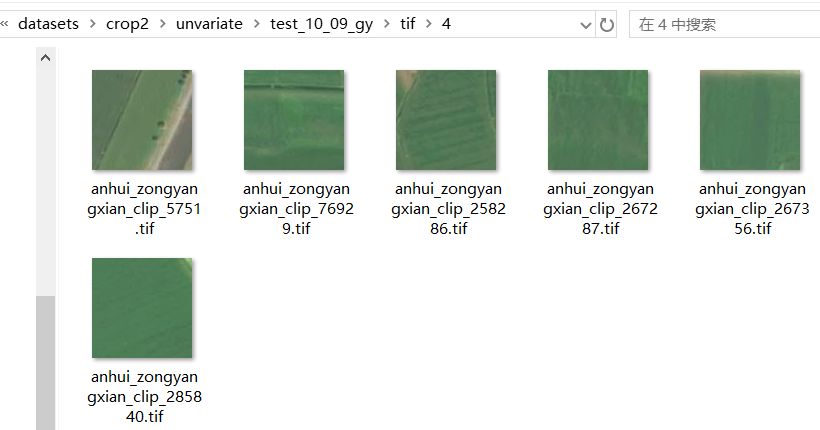


1. Winter wheat

2. Small parcel input sample of k-SPICE_1_


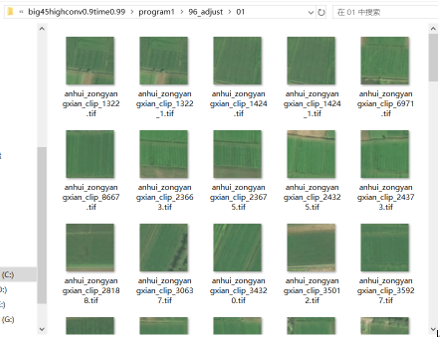


1. Winter wheat


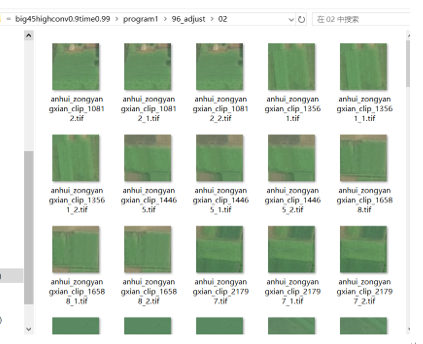


1. Winter rapeseed

3. micro parcel input sample of k-SPICE_2_


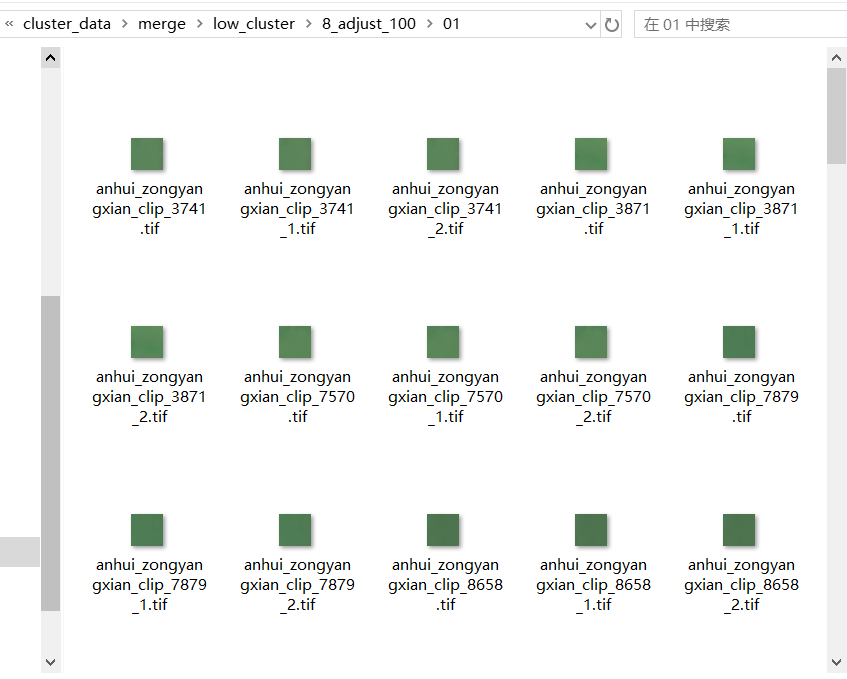


(a)Winter wheat


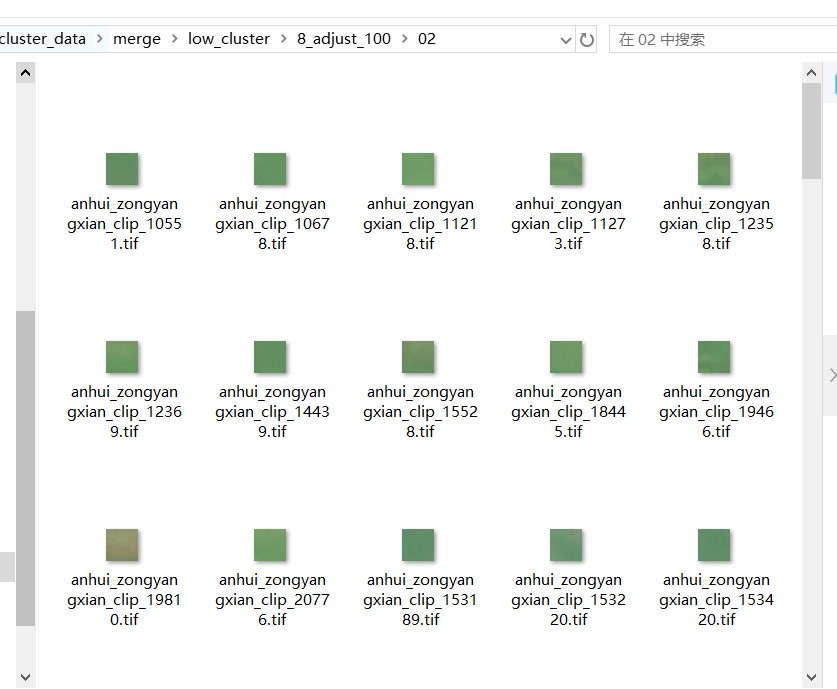


(b)Winter rapeseed
