# Supplementary material for: A precise spatiotemporal fusion crop classification framework based on parcels
Source: Sci Rep. 2025 Jun 1;15:19208. doi: 10.1038/s41598-025-03351-7 (PMC12127452; doi:10.1038/s41598-025-03351-7)

1. The output of KI in arcmap


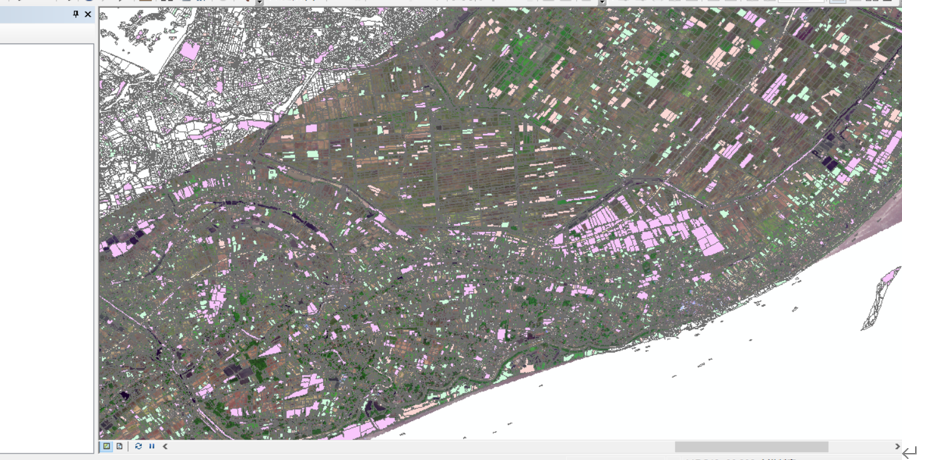


2. The output of k-SPICE_1_ in arcmap(small parcels)


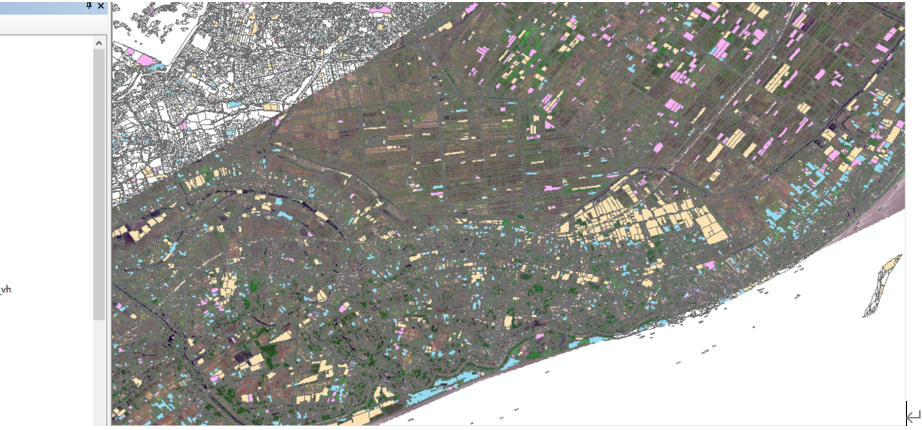


3. The output of k-SPICE_2_ in arcmap (micro parcels)


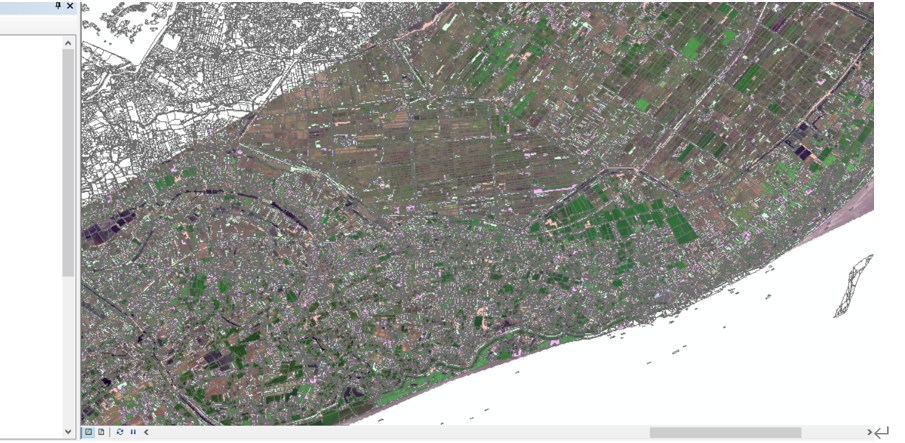


4. The output of k-SPICE_2_ in arcmap (all parcels)


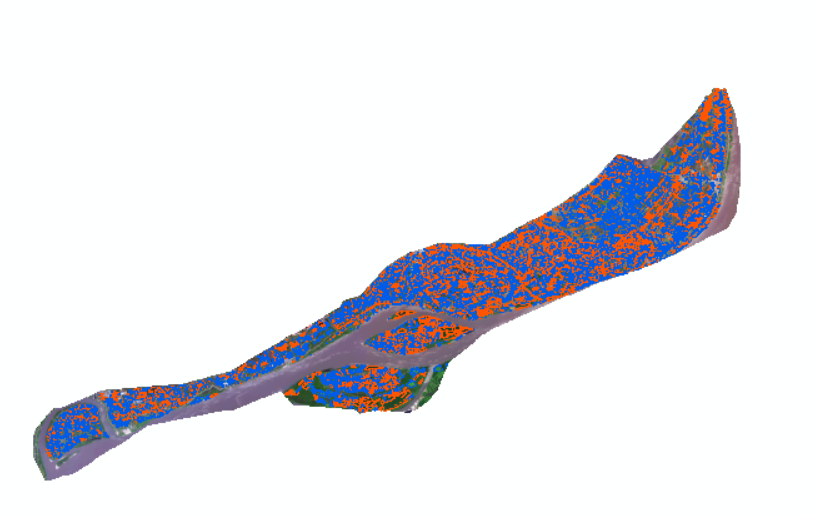

Supplement: Supplementary file 16 — Supplementary Information 16. [file 41598_2025_3351_MOESM16_ESM.docx]
